# A novel MADM model integrating hybrid information for evaluating the development prospects of urban new energy vehicles

**DOI:** 10.1371/journal.pone.0314026

**Published:** 2025-01-28

**Authors:** Yanlong Dong, Donghui Wang, Fanlong Zeng, Yongzheng Zhang

**Affiliations:** 1 School of Foreign Studies, Yiwu Industrial & Commercial College, Yiwu, Jinhua, Zhejiang, China; 2 Business School, University of Shanghai for Science and Technology, Shanghai, China; Opole University of Technology: Politechnika Opolska, POLAND

## Abstract

As an effective approach to mitigating urban environmental issues, New Energy Vehicles (NEVs) have become a focal point of research regarding their current development status and future prospects in China. Addressing the significant disparities in the development of the NEVs industry across different cities, this study focuses on ten typical Chinese cities and develops a novel multi-attribute decision-making (MADM) framework to evaluate the prospects of NEVs promotion in these cities. The study first establishes a comprehensive indicator system that covers key dimensions such as economy, policy support, infrastructure, technological innovation, and environment, encompassing five different types of evaluation information. This system incorporates five different types of evaluation information: exact numbers, interval numbers, triangular fuzzy numbers, hesitant fuzzy numbers, and probabilistic linguistic term sets (PLTS), enhancing the framework’s ability to handle diverse data types. Subsequently, the improved entropy (IEntropy) weight method is employed to determine the objective weights of the evaluation indicators. These objective weights are then integrated with the Vlsekriterijumska Optimizacija I Kompromisno Resenje (VIKOR) method, facilitating a structured group decision-making approach that synthesizes hybrid evaluation information. Based on modular thinking, hybrid evaluation information is synthesized to evaluate and rank the NEVs development prospects of each city. Sensitivity analysis and comparative analysis further demonstrate the robustness and reliability of the proposed MADM framework. The ranking results indicate that Shanghai and Guangzhou lead in NEVs promotion, while cities like Harbin and Zhengzhou lag behind. Based on these findings, the study proposes targeted policy recommendations to promote the sustainable development of the NEVs industry in major Chinese cities.

## 1. Introduction

China joined the Paris Agreement on climate change in 2016 and has since contributed significantly to global climate change efforts [1]. The Chinese government has formulated "carbon peak" and "carbon neutrality" plans across various sectors, aiming to effectively implement greenhouse gas emission targets. According to data released by the China Association of Automobile Manufacturers, in 2023, China’s automobile production and sales both exceeded 30 million units. Faced with such a large increase and stock of automobiles, the automotive industry’s carbon emission pressure is immense. Therefore, promoting the development of green urban transportation to alleviate carbon emission pressure has become an important government objective.

The development of NEVs is a strategic measure by the Chinese government to promote green urban transportation. The NEVs discussed here refer to pure electric vehicles, plug-in hybrid vehicles (including extended-range vehicles), and fuel cell vehicles that are eligible for green license plates and enjoy purchase tax exemptions as designated by the Chinese government. Existing research often explores NEVs development from multiple perspectives, including technological innovation by enterprises, government policy formulation, market promotion, and infrastructure construction. For instance, Sun et al. [2] found that to accelerate NEVs promotion, enterprises should increase their R&D investment in patents for pure electric and hybrid vehicles. Yang et al. [3] studied the impact of China’s fiscal and tax policies on technological innovation in NEVs enterprises. Guo et al. [4], starting from the psychology of consumer purchasing behavior, proposed intervention strategies for NEVs promotion. Wang et al. [5] proposed a new site selection model for NEVs infrastructure construction, while Guo et al. [6] explored the optimal configuration of NEVs and traditional fuel vehicles in cities.

In November 2020, the Chinese government released the "New Energy Vehicle Industry Development Plan (2021–2035)," setting a goal of achieving a 40% market penetration rate for NEVs by 2030. This plan aims to promote low-carbon, environmentally friendly transportation and drive the high-quality, sustainable development of the NEVs industry. Along with this plan, major Chinese cities have also introduced NEVs promotion policies. However, the development prospects of NEVs vary significantly among different cities. These differences are driven by variations in economic conditions, market demand, infrastructure development, and government policy support. Therefore, evaluating the development prospects of NEVs in these cities is crucial for cities to grasp the progress of the NEVs industry, optimize strategies, improve resource allocation efficiency, and achieve the "dual carbon" goals.

Currently, research on the evaluation of NEVs promotion prospects is relatively scarce. While studies focus on various factors affecting NEVs, there is a lack of a comprehensive evaluation framework that addresses the diverse and complex information needed for assessing NEVs development in different cities. To fill this gap, this paper aims to develop a new Multi-Attribute Decision-Making (MADM) method to evaluate NEVs promotion prospects across different cities, specifically by integrating the improved IEntropy weight method with the Mo-RVIKOR method. This approach not only refines the weight determination process but also offers a decision-making framework tailored to the unique challenges of urban NEVs development, allowing for a more comprehensive and rational evaluation of NEVs in different urban contexts.

MADM, as an effective evaluation tool, provides policymakers and researchers with a quantitative analysis method. At present, MADM theories and methods have been well-developed and widely applied in fields such as project management [7], financial investment [8], environmental management [9], medical decision-making [10], and supply chain management [11]. In the transportation sector, MADM methods have also been extensively used to support sustainable development and strategic decision-making. For example, Fang et al. [12] employed a complex intuitionistic fuzzy MADM approach to identify the most eco-friendly transportation mode by balancing environmental, cost, and operational factors. Hasan et al. [13] developed an MADM-based framework that integrates stakeholder engagement, lifecycle cost, and environmental impact assessments for sustainable road transport projects. Saxena et al. [14] used a fuzzy analytical hierarchy process to identify key barriers to the electrification of urban freight in India, helping prioritize policy interventions. Additionally, Aiello et al. [15] applied a TOPSIS-based MADM framework to support zero-emission fleet renewal decisions, addressing both environmental and economic goals under different scenarios. These studies demonstrate the effectiveness of MADM approaches in enhancing sustainability, optimizing transportation systems, and addressing barriers to green technology adoption.

However, in the context of evaluating the development prospects of urban NEVs, the development of MADM methods for hybrid evaluation information is still insufficient. The evaluation of urban NEVs development prospects involves multiple dimensions. In the actual evaluation process, indicator evaluation information is often complex, ambiguous, uncertain, and diverse. Additionally, different evaluators have varying preferred evaluation information and methods, leading to indicator information being expressed in various forms such as interval numbers, semantic values, and fuzzy numbers. Therefore, it is meaningful to study how to handle evaluation information and provide decision-making solutions when indicator information appears in multiple forms in the decision matrix. For such hybrid evaluation information, scholars often convert them into a single type of information for comparison and ranking. For example, Lin et al. [16] converted exact numbers, interval numbers, fuzzy numbers, and linguistic variables in a hybrid MADM matrix into unified Z-numbers. Yang et al. [17], when dealing with hybrid evaluation information, converted it into intuitionistic fuzzy numbers with varying degrees of hesitation and used an improved TOPSIS method to evaluate the comprehensive benefits of intelligent substation construction projects. Zeng et al. [18] proposed a new method for solving hybrid MADM problems by introducing the closeness to positive ideal solutions and a compromise-based dynamic weight decision-making method.

Due to the complexity, uncertainty, and diversity of real-world problems and various types of information, this paper expands the types of evaluation indicators for urban NEVs promotion to include exact numbers, interval numbers, triangular fuzzy numbers, hesitant fuzzy numbers, and probabilistic linguistic term sets (PLTS). If all five types of information are converted into a single type, as per existing research methods, it may lead to information loss and complex calculations. To address these issues, Lourenzutti et al. [19] proposed the modular thinking approach, which classifies indicators based on their characteristics, processes each type of evaluation information independently in a separate module, and then integrates them. This paper combines the VIKOR method with modular thinking, proposing the Mo-RVIKOR method for ranking candidate solutions. When indicator evaluation information is hybrid, the evaluation information is divided into independent modules based on indicator characteristics. The positive and negative ideal solutions for each indicator are obtained without converting the various types of hybrid information. The maximum group benefit and minimum individual regret for each solution are then calculated, followed by the comprehensive indicator calculation for ranking the solutions.

Additionally, since the traditional VIKOR method does not involve weight determination, and the weights given by experts often carry subjective bias and uncertainty, selecting a reasonable weight determination method is particularly important. Common objective weight determination methods include goal programming, entropy weight method, deviation maximization method, and solution maximization method. The entropy weight method is a common method for determining indicator weights in MADM problems. However, Deng et al. [20] pointed out that in extreme cases (e.g., when the entropy value approaches 1), small differences in entropy values among different indicators can cause significant differences in entropy weights. To address this issue, this paper proposes an improved entropy weight method, the IEntropy weight method. IEntropy method optimizes the calculation of entropy values within the traditional entropy method by introducing new adjustment factors. These factors dynamically adjust according to the specific distribution of indicator data, thus more reasonably addressing indicators with small variations but high importance. By using this approach, the issue of uneven weight distribution caused by small differences in entropy values among indicators is effectively mitigated. This enhanced method not only strengthens the stability and reliability of the entropy method when dealing with extreme data but also improves the scientific and rational allocation of weights. The IEntropy method has been applied within the Mo-RVIKOR decision framework, enhancing the model’s accuracy and efficiency in handling multi-type evaluation information. In the Mo-RVIKOR method, the weights determined by the IEntropy method are combined with a composite evaluation index of the indicators, enabling a more accurate arrangement and selection of the best decision options.

In summary, this study is divided into seven sections. Section 1 introduces the research background, objectives, and significance of evaluating urban NEVs development prospects. Section 2 presents the study area, indicator system, and data sources used in the analysis. Section 3 provides an overview of the mathematical definitions and computational formulas for the five types of evaluation information referenced in this study. Section 4 introduces the IEntropy-Mo-RVIKOR evaluation framework developed to assess the development prospects of urban NEVs. Section 5 demonstrates a case study analysis, comparing model results from multiple perspectives. Section 6 discusses NEVs promotion in ten representative cities in China, offering insights into regional variations. Finally, Section 7 concludes with key findings and provides recommendations for NEVs development.

## 2. Study area, indicator system, and data sources

### 2.1. Overview of the study area

According to data from the International Energy Agency, global electric vehicle (EV) sales reached nearly 14 million units in 2023, representing 18% of total vehicle sales. China accounted for approximately 60% of these sales, followed by Europe (25%) and the United States (10%). These three regions collectively represent 65% of global automobile sales, indicating that EV sales remain geographically concentrated relative to traditional vehicles. In China, the NEVs industry benefits from substantial government intervention, with policies that include R&D support, consumer tax incentives, license facilitation for NEVs in major cities, and foreign investment encouragement, exemplified by Tesla’s factory in Shanghai. This government-driven approach stands in contrast to the more market-oriented frameworks seen in Europe and the U.S., where government involvement in NEVs development is generally less direct.

**Fig 1:**
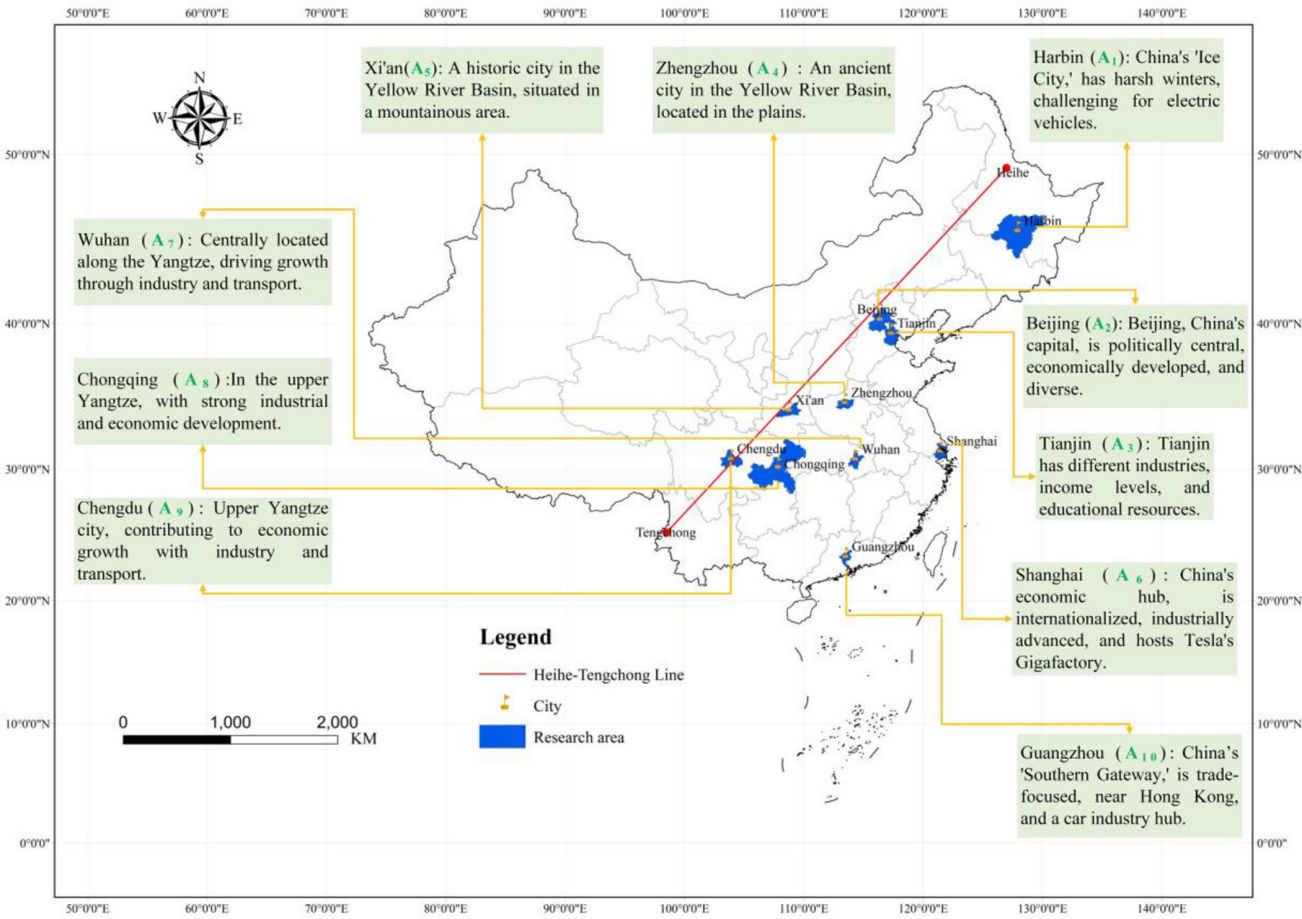
Map of study areas. Reprinted background map from the National Catalogue Service for Geographic Information (www.webmap.cn) under a CC BY license, with permission from the Ministry of Natural Resources of China, original copyright 2019.

This study selects ten representative cities in China as the research subjects (Fig 1). These ten cities are all located east of the "Heihe-Tengchong Line," an imaginary line that marks a significant demographic divide in China [21]. Southeast of this line, which covers 36% of China’s land area, resides 96% of the country’s population (according to the Fifth National Population Census). The cities, arranged approximately in a latitudinal direction from north to south, include Harbin (*A*_*1*_), Beijing (*A*_*2*_), Tianjin (*A*_*3*_), Zhengzhou (*A*_*4*_), Xi’an (*A*_*5*_), Shanghai (*A*_*6*_), Wuhan (*A*_*7*_), Chongqing (*A*_*8*_), Chengdu (*A*_*9*_), and Guangzhou (*A*_*10*_). These cities account for about 2% of China’s total area but host around 13% of the national permanent population, placing significant pressure on the environment and resources. Additionally, these cities have notable geographical, economic, and cultural representativeness, making the study of their NEVs development prospects crucial for addressing urban environmental issues and alleviating resource constraints.

Harbin is the northernmost provincial capital in China, with the lowest annual average temperature. From a technical perspective, the suitability of the environment for NEVs, especially electric vehicles, is not high. Beijing, as the capital of China, is a political center with a robust economic foundation. It is the hub of the "Beijing-Tianjin-Hebei Economic Integration" and has a population exceeding 20 million, with per capita income among the highest in the country. Tianjin, adjacent to Beijing, is a directly governed municipality with Tianjin Port, a key node for land and sea transportation. Economically and industrially, Tianjin lags behind Beijing. Zhengzhou and Xi’an are provincial capitals in the northern Yellow River region, serving as central cities in central and northwestern China, respectively. The Yellow River basin, as the birthplace of Chinese civilization, has a relatively conservative social culture influenced by historical and cultural factors. Agricultural culture has historically dominated, and commercial civilization and market openness are less developed compared to southern regions.

Shanghai, Wuhan, Chongqing, and Chengdu are four cities located along the Yangtze River. Shanghai, at the mouth of the Yangtze River, is a directly governed municipality and is considered China’s economic center, with a population nearing 25 million and one of the highest per capita incomes in the country. Tesla’s China Gigafactory is located in Shanghai. Wuhan, a provincial capital in central China, is a comprehensive transportation hub with a high level of industrial development. Chongqing, also a directly governed municipality, and Chengdu, a provincial capital, together form an urban agglomeration playing a significant role in economic development, population concentration, and urbanization in southwestern China. Guangzhou, a provincial capital, leads in comprehensive economic development indicators such as industrial development, market openness, population density, international trade, and transportation.

For this study, focusing on China’s NEVs market—representing a significant share of global EV sales—provides valuable context for examining regional differences in NEVs adoption. However, given the substantial differences in market structures, economic conditions, and consumer behaviors between China and other regions, direct comparisons with foreign markets may require a tailored research framework to accommodate variations in governance and economic settings. Future studies will aim to expand this research by incorporating cross-national comparisons, thereby assessing the adaptability of our framework across different governance and market contexts.

Through a comprehensive analysis of the NEVs development prospects in these ten cities, this study aims to provide further insights and practical strategies for the development of the NEVs industry in China and globally.

### 2.2. Indicator system

Evaluating the development prospects of urban NEVs forms an integral part of macroeconomic research. This study, inspired by the macro-analysis framework known as the PESTE model [22], focuses on five key dimensions: policy support, economic and market conditions, social infrastructure, technological innovation, and environmental and climatic factors. This section reviews relevant literature based on these dimensions.

In the economic and market dimension, selecting indicators such as per capita income, total GDP, population size, market penetration rate, and NEVs production penetration rate provides a foundational understanding of the economic potential for NEVs development in a city. Per capita income directly correlates with consumers’ purchasing power for NEVs, while total GDP reflects the overall economic scale, indicating a city’s financial capacity to support NEVs infrastructure and incentives. Population size relates to the potential consumer base for NEVs, which can directly shape market demand. Market penetration rate reflects the degree to which NEVs are accepted in the city’s automotive market, providing insight into local adoption levels [23, 24]. These economic indicators are crucial for forecasting a city’s NEVs adoption and highlight the economic benefits of transitioning to sustainable transportation solutions.

Policy support plays a pivotal role in promoting NEVs adoption. Indicators such as purchase subsidy intensity, license policy, green finance, and tax incentives are vital for assessing how government interventions make NEVs more accessible and affordable. For example, purchase subsidies and tax exemptions directly reduce the financial burden on consumers, making NEVs a more attractive option. License policies and green finance initiatives, which include financial incentives for green projects, further support NEVs by addressing cost and accessibility barriers [25, 26]. By including these policy indicators, this study aims to capture how government policies can foster an enabling environment for NEVs and accelerate the shift toward sustainable transportation.

Infrastructure is a critical factor in supporting NEVs development and increasing consumer confidence in NEVs usage. The presence of adequate charging infrastructure, represented by the number of battery swap and charging stations, determines the accessibility and convenience of NEVs, directly impacting consumers’ willingness to adopt NEVs. The number of service outlets reflects the city’s ability to maintain a robust service network, which supports ongoing maintenance and repairs, thereby enhancing user confidence. Furthermore, indicators like traffic congestion and public transportation coverage provide insight into the city’s broader transportation environment, affecting the practicality of NEVs as part of an integrated urban transport system [27, 28]. This infrastructure is vital for supporting NEVs adoption as part of a city’s comprehensive approach to sustainable transport.

Technological innovation in NEVs plays a crucial role in improving vehicle performance, lowering production costs, and ultimately expanding NEVs accessibility. Indicators such as R&D investment and the urban innovation index measure a city’s commitment to advancing NEVs-related technologies. Higher R&D investments contribute to advancements in battery life, charging speeds, and overall vehicle performance, making NEVs more competitive with traditional vehicles. By focusing on these indicators, can evaluate the technological readiness of cities and their potential to support and advance NEVs technology, which is essential for long-term NEVs market growth [29].

Environmental and climatic factors are essential for assessing the sustainability and feasibility of NEVs in different urban contexts. Indicators such as air quality and carbon emissions measure the potential environmental benefits that NEVs can bring to a city. For instance, improving air quality and reducing carbon emissions are key goals of NEVs adoption, as they directly support a city’s environmental sustainability objectives. Temperature suitability is included because extreme temperatures can impact battery performance, thus affecting NEVs efficiency and user satisfaction [30]. By incorporating these environmental indicators, can assess the broader sustainability impact of NEVs and their role in meeting urban climate goals.

**Fig 2:**
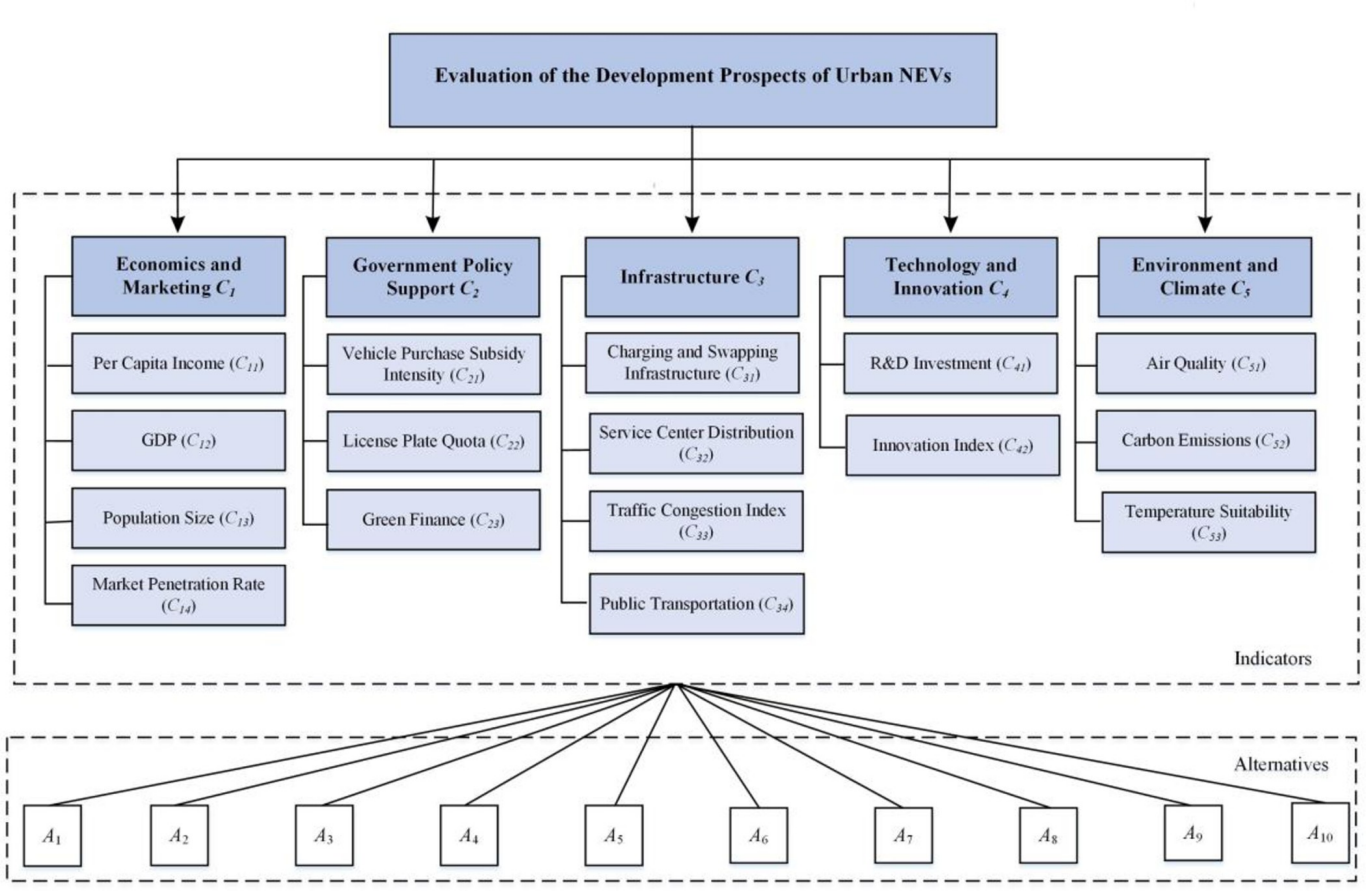
Evaluation index system for the development prospects of NEVs.

Based on the above literature review, the evaluation indicators for the development prospects of urban NEVs are summarized in Table 1. To ensure the availability of evaluation indicator data and enhance the rationality of the evaluation indicators, a brainstorming session was organized. The results led to the exclusion of four indicators: production penetration rate, industrialization level, tax incentives, and social cultural openness. The production penetration rate is highly correlated with the market penetration rate, potentially leading to redundant evaluation information. Considering the well-developed logistics system in China, NEVs market sales can achieve smooth cross-city distribution, thus the industrialization level of NEVs has a minimal impact on the urban NEVs market. Tax incentives, being national policies, do not vary between cities. The social cultural differences in China are mainly reflected in the regional differences between the north and south, which often influence local population behavior choices [31]. However, social cultural openness is difficult to quantify. Based on these considerations, the final evaluation indicator system was constructed (as shown in Fig 2), aiming to provide a comprehensive and rational analysis framework for urban NEVs development.

**Table 1:** Summary of evaluation index system for the development prospects of urban NEVs.

Primary Indicators	Secondary Indicators	Indicator Meaning
Economics and Marketing	Per Capita Income	The average income per resident in the city, reflecting the purchasing power for NEVs.
	Total GDP	The total gross domestic product of the city, measuring the overall economic scale.
	Population Size	Significantly related to the size of the consumer group in the city, directly shaping the market potential for NEVs.
	Market Penetration Rate	The proportion of NEVs in the city’s car market, indicating their penetration in the overall car market.
	Production Penetration Rate	The proportion of NEVs in the city’s car production, reflecting the city’s involvement in NEVs production.
	Industrialization Level	Reflects the city’s industrial foundation, closely related to the development of the NEVs industry.
Government Policy Support	Purchase Subsidy Intensity	The level of economic incentives provided by the government for purchasing NEVs.
	License Policy	Policies regarding the issuance and management of licenses for NEVs.
	Green Finance	Government financial policies supporting green energy and sustainable development.
	Tax Incentives	Tax reductions or exemptions provided by the government for NEVs and related industries.
Social and Infrastructure	Cultural Openness	The acceptance level of NEVs among the city’s permanent residents.
	Number of Battery Swap and Charging Stations	The number of facilities in the city providing charging and battery swap services for NEVs.
	Number of Service Outlets	The number of service outlets for NEVs maintenance and related services.
	Traffic Congestion Index	Measures the level of traffic congestion in the city, affecting the use and promotion of NEVs.
	Public Transportation	The development and coverage of the city’s public transportation system.
Technology and Innovation	R&D Investment	Reflects the city’s level of technological research and development.
	Urban Innovation Index	Measures the city’s level of innovation, related to technological and industrial innovation in NEVs.
Environment and Climate	Air Quality	Measures the purity of the city’s air, related to the environmental benefits of NEVs.
	Carbon Emissions	The total carbon emissions produced by the city, impacting the environmental benefits of NEVs.
	Temperature Suitability	Measures whether the city’s temperature is suitable for the use and charging of NEVs.

As we know, Economic and marketing (*C*_*1*_) data can often be obtained from specific financial reports and market research, such as income, sales volume, and market share. These data are highly precise and can be accurately represented by precise numbers, which facilitates quantitative analysis and comparison.

Government policy support (*C*_*2*_) is often difficult to predict precisely, and the effects of policy implementation can vary within a range. For example, the rate of investment growth under policy guidance and the impact of tax incentives can fluctuate. Using interval numbers can better express this uncertainty and the expected range of variation.

The assessment of infrastructure (*C*_*3*_) development often involves factors with high uncertainty, such as public satisfaction and predictions of future infrastructure needs. Triangular fuzzy numbers can effectively express this uncertainty by describing information through a triplet (most likely value, minimum value, maximum value).

The uncertainty of technological development and innovation (*C*_*4*_) speed, along with various possible development paths, make hesitant fuzzy numbers a suitable choice. This type of data allows evaluators to express hesitation and uncertainty about multiple possible values for the same indicator, reflecting a diverse assessment of technological potential and innovative outcomes.

Environmental and climate (*C*_*5*_) change data often involve high levels of uncertainty and complexity. Probabilistic linguistic term sets can combine qualitative assessments and quantitative probabilities, effectively describing potential impacts of climate change on ecosystems, and varying effects of environmental policies, among others. In this manner, evaluators can use linguistic terms to express opinions and assign a probability to each term to indicate the likelihood of different outcomes.

### 2.3. Data sources

The primary data for this study comes from the 2023 statistical yearbooks, statistical bulletins, and government websites of major cities. All data pertains to the year 2022 across the various indicators for each city. The apparent reference to "2023" in certain data points refers to the naming convention of the source publications, which use the release year (2023) to title the reports, even though the data itself reflects the previous year (2022). No missing data issues were encountered, as the study adhered strictly to the principle of data availability when constructing the indicator system.

The specific sources are as follows:

Commuting Peak Congestion Index: This data is sourced from Baidu Map’s "2023 China Urban Transportation Report."

Rail Transit Operation Data: Sourced from the Ministry of Transport of the People’s Republic of China.

Infrastructure Construction Data: Obtained from Baidu Map search results.

Shanghai’s Penetration Rate: Comes from the China Passenger Car Association’s "December 2023 Shanghai Automobile Market Analysis."

New Energy Vehicle Sales Penetration Rate for 2022: Derived from the Gasgoo Auto Research Report.

Green Finance Data: Obtained from the Wind database.

R&D Investment: Provided by the National Bureau of Statistics of China.

Urban Innovation Index: Sourced from the "China Urban Science and Technology Innovation Development Report (2022)."

Annual Air Pollution Days and Temperature Data: Retrieved from the China Meteorological Data Service Center.

Carbon Emissions Data: Calculated based on the carbon emission estimation method by Wu et al. [32], using centralized heating data from the "2022 China Urban Construction Statistical Yearbook."

## 3. Preliminaries

This study involves five types of evaluation information: exact numbers, interval numbers, triangular fuzzy numbers, hesitant fuzzy numbers, and PLTS. The basic concepts and operational rules for these types of evaluation information are introduced as follows.

### 3.1. Precise numbers

When the evaluation information of attribute *C*_*j*_ is numerical, and there is inconsistency in units, standardization of the information is required. Let *y*_*ij*_ be the initial evaluation information, and ${\bar{y}}_{ij}$ be the standardized information. The calculation expressions are as follows [33]:

When the indicator is cost-type:

y¯ij=1yij∑i=1n(1yij)2 (1)

When the indicator is benefit-type:

$${\bar{y}}_{ij}=\frac{y_{ij}}{\sqrt{\sum_{i=1}^{n}{\left(y_{ij}\right)}^{2}}}$$ (2)

Example 1. A certain project has two options, Plan A and Plan B, with respective costs of 6 million yuan and 5 million yuan. These costs are classified as cost-type, and according to Formula 1, they can be standardized as follows: A¯=(16)(16)2+(15)2=0.64, B¯=(15)(16)2+(15)2=0.768.

### 3.2. Interval numbers

An interval number is a way to represent uncertainty by specifying a range between two values: a lower bound and an upper bound. This approach is particularly useful when the exact value of something is not known but is expected to lie within a specific range. Next, we introduce the concept of interval numbers.

Definition 1 [33]: Let *R* be the set of real numbers. An interval number can be represented in the following form: $\tilde{r}=[r^{L},r^{U}]=\{r^{L}\leq r\leq r^{U},r\in R\}$, where *r*^*L*^ and *r*^*U*^ are the lower and upper bounds of the interval number, respectively. Specifically, when *r*^*L*^ = *r*^*U*^, the interval number $\tilde{r}$ degenerates into a precise number.

Definition 2 [33]: Let $\tilde{r}=[r^{L},r^{U}]=\{r^{L}\leq r\leq r^{U},r\in R\}$ be an interval number. Its score function is defined as:

$$S(\tilde{r})=\frac{r^{L}+r^{U}}{2}$$ (3)

Definition 3 [33]: If ${\tilde{r}}_{1}=\left[r_{1}^{L},r_{1}^{U}\right]$ and ${\tilde{r}}_{2}=\left[r_{2}^{L},r_{2}^{U}\right]$ are two interval numbers, their Euclidean distance is defined as:

$$d({\tilde{r}}_{1},{\tilde{r}}_{2})=\sqrt{\frac{1}{2}[{\left(r_{1}^{L}-r_{2}^{L}\right)}^{2}+{\left(r_{1}^{U}-r_{2}^{U}\right)}^{2}]}$$ (4)

If ${\tilde{r}}_{1}=\left[r_{1}^{L},r_{1}^{U}\right]$ and ${\tilde{r}}_{2}=\left[r_{2}^{L},r_{2}^{U}\right]$ are two interval numbers, their comparison method is: Let $r_{1}=\frac{r_{1}^{L}+r_{1}^{U}}{2}$, $r_{2}=\frac{r_{2}^{L}+r_{2}^{U}}{2}$, if *r_1_* > *r*_2_, then ${\tilde{r}}_{1}>{\tilde{r}}_{2}$; if $r_{1}=r_{2}$, then ${\tilde{r}}_{1}={\tilde{r}}_{2}$; if *r*_1_ < *r*_2_, then ${\tilde{r}}_{1}<{\tilde{r}}_{2}$.

Example 2. A certain project has two options, Plan A and Plan B. Plan A is expected to generate profits between 25% and 35%, while Plan B is expected to generate profits between 20% and 30%. By calculating their score functions using Formula 3, the results can be obtained as follows.


$$S(\bar{A})=\frac{0.25+0.35}{2}=0.3,S(\bar{B})=\frac{0.2+0.3}{2}=0.25$$

### 3.3. Triangular fuzzy numbers

A triangular fuzzy number is a way to represent uncertainty or fuzziness using a three-part representation: a minimum value, a most likely value, and a maximum value. This method is particularly useful for describing things that cannot be precisely defined but have a certain range and trend. Next, we introduce the concept of triangular fuzzy numbers.

Definition 4 [34]: Let $\tilde{a}=(a^{L},a^{M},a^{U})$ and $\tilde{b}=(b^{L},b^{M},b^{U})$ be two triangular fuzzy numbers. The addition of triangular fuzzy numbers is defined as:

$$\tilde{a}⊕\tilde{b}=(a^{L},a^{M},a^{U})⊕(b^{L},b^{M},b^{U})=(a^{L}+b^{L},a^{M}+b^{M},a^{U}+b^{U})$$ (5)

Definition 5 [34]: The multiplication of triangular fuzzy numbers is defined as:

$$t⊗\tilde{a}=t⊗(a^{L},a^{M},a^{U})=(ta^{L},ta^{M},ta^{U})$$ (6)

Usually, when calculating the distance between two triangular fuzzy numbers, the traditional Euclidean distance is used. Relative preference relation analysis offers advantages over traditional Euclidean distance in calculating the distance of triangular fuzzy numbers, better avoiding the loss of evaluation information [34].

Definition 6 [34]: Let $\tilde{a}=(a^{L},a^{M},a^{U})$ and $\tilde{b}=(b^{L},b^{M},b^{U})$ be two triangular fuzzy numbers. The relative preference relation of $\tilde{a}$ to $\tilde{b}$ is defined as:

$$\mu_{p}(\tilde{a},\tilde{b})=\frac{1}{2}\left(\frac{\left(a^{L}-b^{U}\right)+2\left(a^{M}-b^{M}\right)+\left(a^{U}-b^{L}\right)}{2\|T\|}+1\right)$$ (7)

Where $\left\|T\right\|=\left\{\begin{array}{l} \frac{\left(t_{L}^{+}-t_{U}^{-})+2(t_{M}^{+}-t_{M}^{-})+(t_{U}^{+}-t_{L}^{-}\right)}{2},\;\text{when}t_{L}^{+}-t_{U}^{-}\geq 0\\ \frac{\left(t_{L}^{+}-t_{U}^{-})+2(t_{M}^{+}-t_{M}^{-})+(t_{U}^{+}-t_{L}^{-}\right)}{2}+2(t_{U}^{-}-t_{L}^{+}),\;\text{when}t_{L}^{+}-t_{U}^{-}<0 \end{array}\right.\;$, $t_{L}^{+}=\max\left\{a^{L},b^{L}\right\}$, $t_{M}^{+}=\max\left\{a^{M},b^{M}\right\}$, $t_{U}^{+}=\max\left\{a^{U},b^{U}\right\}$, $t_{L}^{-}=\min\left\{a^{L},b^{L}\right\}$, $t_{M}^{-}=\min\left\{a^{M},b^{M}\right\}$, $t_{U}^{-}=\min\left\{a^{U},b^{U}\right\}$.

If $\tilde{a}=(a^{L},a^{M},a^{U})$ and $\tilde{b}=(b^{L},b^{M},b^{U})$ are two triangular fuzzy numbers, their comparison method is: Let $a=\frac{a^{L}+4a^{M}+a^{U}}{6}$, $b=\frac{b^{L}+4b^{M}+b^{U}}{6}$. If *a*>*b*, then $\tilde{a}>\tilde{b}$; if *a* = *b*, then $\tilde{a}=\tilde{b}$; if *a*<*b*, then $\tilde{a}<\tilde{b}$.

Definition 7 [34]: If $\tilde{a}=(a^{L},a^{M},a^{U})$ is a triangular fuzzy number, its score function is defined as:

$$S(\tilde{a})=\frac{a^{L}+a^{M}+a^{U}}{3}$$ (8)

Example 3. A certain project has two options, Plan A and Plan B. Plan A is expected to be completed in as early as 18 months, as late as 28 months, with the most likely completion time being 23 months. Plan B is expected to be completed in as early as 20 months, as late as 30 months, with the most likely completion time being 24 months. By calculating their score functions using Formula 8, the results can be obtained as follows.


$$S(\bar{A})=\frac{18+23+28}{3}=23,S(\bar{B})=\frac{20+24+30}{3}=24.6.$$

### 3.4. Hesitant fuzzy numbers

Hesitant fuzzy numbers are a way to represent uncertainty when there are multiple possible values for a given parameter, and the decision-maker is unsure about which one to choose. This approach is particularly useful when there is hesitation among several possible values rather than a single estimate.

Definition 6 [35]: Let *T*be a given ordered set. A hesitant fuzzy set *H* defined on *T* is a function that maps *T* to a subset of the interval [0,1]. Mathematically, it can be expressed as:

$$H=\{(t,h_{H}(t))|t\in T\}$$ (9)

Where *h*_*H*_(*t*) is a set of several different real numbers in the interval [0,1]. These numbers represent the degrees to which *t* belongs to the hesitant fuzzy set *H*, and they are the basic elements of the hesitant fuzzy set *H*. A hesitant fuzzy number ℎ is one such set, where $h=h_{H}(t)=\{\gamma |\gamma \in h_{H}(t)\}=H\{\gamma^{1},\gamma^{2},\cdots ,\gamma^{l}\}$, $\gamma^{\lambda }\in [0,1],\lambda =1,2,\cdots ,l$ and *l* denotes the number of elements in the hesitant fuzzy number *h*.

Definition 7 [35]: For any three hesitant fuzzy elements *h*, *h*_*1*_ and *h*_*2*_, their basic arithmetic operations are defined as follows (where, *θ* > 0):

1. $h_{1}\cup h_{2}=H\{\max(\gamma_{1},\gamma_{2})|\gamma_{1}\in h_{1},\gamma_{2}\in h_{2}\}$;
2. $h_{1}\cap h_{2}=H\{\min(\gamma_{1},\gamma_{2})|\gamma_{1}\in h_{1},\gamma_{2}\in h_{2}\}$;
3. $\theta h=H\{(1-{\left(1-\gamma \right)}^{\theta }\}|\gamma \in h\}(\theta >0)$;
4. $h^{\theta }=H\{\gamma^{\theta }|\gamma \in h\}(\theta >0)$;
5. $h^{c}=H\{(1-\gamma )|(\gamma \in h)\}$;
6. $h_{1}⊕h_{2}=H\{\gamma_{1}+\gamma_{2}-\gamma_{1}\gamma_{2}|\gamma_{1}\in h_{1},\gamma_{2}\in h_{2}\}$;
7. $h_{1}⊗h_{2}=H\{\gamma_{1}\gamma_{2}|\gamma_{1}\in h_{1},\gamma_{2}\in h_{2}\}$.

Definition 8: For two hesitant fuzzy numbers $h_{1}=H\{\gamma_{1}^{\lambda }|\lambda =1,2,\cdots ,l_{1}\}$ and $h_{2}=H\{\gamma_{2}^{\lambda }|\lambda =1,2,\cdots ,l_{2}\}$, assume their elements are arranged in ascending order and have the same number of elements. That is, *l*_1_ = *l*_2_ = *l*, where $\gamma_{1}^{\lambda }$ and $\gamma_{2}^{\lambda }$ represent the λ-th smallest values in $h_{1}$ and *h*_2_, respectively. Then, *h*_1_ ≤ *h*_1_ if and only if $\gamma_{1}^{\lambda }$ ≤ $\gamma_{2}^{\lambda }$, λ = 1,2,…,*l*.

For any two hesitant fuzzy numbers *h*_*1*_ and *h*_*2*_, if they have different numbers of elements, i.e., *l*_1_≠*l*_2_, the hesitant fuzzy number with fewer elements should be extended so that both have the same number of elements. According to the extension rule in [35], this is done as follows:

Definition 9: For a hesitant fuzzy number $h=H\{\gamma_{1}^{\lambda }|\lambda =1,2,\cdots ,l\}$, let λ^+^ and λ^-^ be the maximum and minimum elements of *h*, respectively. Then, $\bar{\lambda }=\eta \lambda^{+}+(1-\eta )\lambda^{-}$ is called a parameterized extended value, where the parameter *η*(0 < *η* < 1) is predetermined by the decision-maker based on their risk preference.

1. When *η* = 1, the extended value $\bar{\lambda }=\lambda^{+}$, In this case, the largest element in the hesitant fuzzy number should be added, and the decision-maker is risk-seeking.
2. When *η* = 0, the extended value $\bar{\lambda }=\lambda^{-}$, In this case, the smallest element in the hesitant fuzzy number should be added, and the decision-maker is risk-averse.
3. When *η* = 1/2, the extended value, in this case, the average of all elements in the hesitant fuzzy number should be added, and the decision-maker is risk-neutral.

Definition 10: Let $h=H\left\{\gamma^{\lambda }|\lambda =1,2,\cdots ,l\right\}$ be a hesitant fuzzy number, then its score function is defined as:

$$S(h)=\frac{1}{l}(r^{1}+r^{2}+\cdots +r^{l})$$ (10)

Drawing on the classical Euclidean distance measure, Xu and Xia [36] proposed the hesitant fuzzy Euclidean distance measure:

$$d_{E}(h_{1},h_{2})=\sqrt{\frac{1}{l}{\sum_{\lambda =1}^{l}\left(\gamma_{1}^{\lambda }-\gamma_{2}^{\lambda }\right)}^{2}}$$ (11)

Example 4. A certain project has two options, Plan A and Plan B. The development team needs to assess the potential risks of two projects, and the experts have shown hesitation in their views on the risks. Expert 1 believes that the risk for Project A could be 19%, Expert 2 believes it could be 25%, and Expert 3 believes it could be 22%. Expert 1 thinks that the risk for Project B could be 22%, Expert 2 believes that the risk for Project A could be 27%, and Expert 3 believes that the risk for Project B could be 25%. By calculating their score functions using Formula 10, the results can be obtained as follows.


$$S(A)=\frac{1}{3}(0.19+0.25+0.22)=0.22,\;S(B)=\frac{1}{3}(0.22+0.27+0.25)=0.247.$$

### 3.5. Probabilistic linguistic term sets

In practical decision-making problems, qualitative indicators such as fluency and comprehension are difficult to evaluate using precise numerical information. In 2016, Pang et al. [37] proposed PLTS, which use a set of linguistic terms along with their corresponding probabilities to represent expert linguistic evaluations. Unlike hesitant fuzzy linguistic term sets, where each element is assigned equal weight, PLTS assigns probabilities to the linguistic terms, reflecting different degrees of preference by experts and avoiding the loss of preference information.

Definition 11 [37]: Let *S* = {*s*_0_,…,*s*_*α*_} be a linguistic term set (LTS), a PLTS can be defined as:

$$L(p)=\{L^{\left(k\right)}(p^{\left(k\right)}){|L^{\left(k\right)}\in S,p}^{\left(k\right)}\geq 0,\;k=1,\;2,\ldots ,\#L(p),\sum_{k=1}^{\#L(p)}p^{\left(k\right)}\leq 1\}$$ (12)

Where, $L^{\left(k\right)}(p^{\left(k\right)})$ denotes that the probability of *L*^(*k*)^ is *p*^(*k*)^, #*L*(*p*) is the number of semantic elements contained in *L*(*p*).

Definition 12 [37]: Let *L*(*p*)_1_ and *L*(*p*)_2_ be two probabilistic linguistic term sets, $L{\left(p\right)}_{1}=\left\{\left.L_{1}^{\left(k\right)}(p_{1}^{\left(k\right)})\right|k=1,2,\ldots ,\#L{\left(p\right)}_{1}\right\}$, $L{\left(p\right)}_{2}=\left\{\left.L_{2}^{\left(k\right)}(p_{2}^{\left(k\right)})\right|k=1,2,\ldots ,\#L{\left(p\right)}_{2}\right\}$. If #*L*(*p*)_1_ > #*L*(*p*)_2_, it is necessary to add #*L*(*p*)_1_ - #*L*(*p*)_2_ semantic elements to the set *L*(*p*)_2_,making the two probabilistic linguistic term sets have the same number of elements. The added semantic elements should be the smallest linguistic terms in the *L*(*p*)_2_, with a probability of 0.

Definition 13 [37]: Let $\sum_{k=1}^{\#L(p)}p^{\left(k\right)}<1$, After normalization, it becomes:

$$\bar{L}(p)=\left\{\left.L^{\left(k\right)}({\bar{p}}^{\left(k\right)})\right|k=1,2,\ldots ,\#L(p)\right\}$$ (13)

Where ${\bar{p}}^{\left(k\right)}=p^{\left(k\right)}/\sum_{k=1}^{\#L(p)}p^{\left(k\right)}$, and *L*^(*k*)^(*p*^(*k*)^) arranged in ascending order.

Let $L{\left(p\right)}_{1}=\{s_{4}(0.3),s_{3}(0.4),s_{2}(0.1)\}$ and $L{\left(p\right)}_{2}=\{s_{4}(0.4),s_{3}(0.5)\}$ be two probabilistic linguistic term sets. According to Definition 12, we obtain $L{\left(p\right)}_{1}=\{s_{2}(0.125),s_{3}(0.5),s_{4}(0.375)\}$。 Since #*L*(*p*)_1_ > #*L*(*p*)_2_, add the smallest linguistic terms *s*_3_ in *L*(*p*)_2_ with a probability of 0, $L{\left(p\right)}_{2}=\{s_{4}(0.4),s_{3}(0.5),s_{3}(0)\}$, according to Definition 13, we obtain $L{\left(p\right)}_{2}=\{s_{3}(0),s_{3}(0.556),s_{4}(0.444)\}$.

Definition 14 [37]: Let $L_{1}^{\left(k\right)}$ and $L_{2}^{\left(k\right)}$ be the *k*-th linguistic terms in *L*(*p*)_1_ and *L*(*p*)_2_, respectively. Let $p_{1}^{\left(k\right)}$ and $p_{2}^{\left(k\right)}$ be the probabilities associated with the linguistic terms $L_{1}^{\left(k\right)}$ and $L_{2}^{\left(k\right)}$, respectively. The distance between *L*(*p*)_1_ and *L*(*p*)_2_ is given by:

$$d\left(L{\left(p\right)}_{1},L{\left(p\right)}_{2}\right)=\sqrt{\sum_{k=1}^{\#L{\left(p\right)}_{1}}{\left(p_{1}^{\left(k\right)}r_{1}^{\left(k\right)}-p_{2}^{\left(k\right)}r_{2}^{\left(k\right)}\right)}^{2}/\#L{\left(p\right)}_{1}}$$ (14)

Where $r_{1}^{\left(k\right)}$ and $r_{2}^{\left(k\right)}$ are the subscripts of the linguistic terms in $L_{1}^{\left(k\right)}$ and $L_{2}^{\left(k\right)}$, respectively.

Definition 15 [37]: Let $L(p)=\{L^{\left(k\right)}(p^{\left(k\right)})$$\left|k=\right.1,2,\ldots ,\#L(p)\}$ be a PLTS, and let *r*^(*k*)^ be the subscript of the linguistic term *L*^(*k*)^, The score function of *L*(*p*) is defined as:

$$S(L(p))=S_{\bar{\alpha }}$$ (15)

Where $\bar{\alpha }=\sum_{k=1}^{\#L(p)}r^{\left(k\right)}p^{\left(k\right)}/\sum_{k=1}^{\#L(p)}p^{\left(k\right)}$. For PLTS *L*(*p*)_1_ and *L*(*p*)_2_, if $S(L{\left(p\right)}_{1})>S(L{\left(p\right)}_{2})$, then *L*(*p*)_1 >_ *L*(*p*)_2_.

Definition 16 [37]: Let $L(p)=\{L^{\left(k\right)}(p^{\left(k\right)})$ $\left|k=\right.1,2,\ldots ,\#L(p)\}$ be a PLTS, *r*^(*k*)^ be the subscript of the linguistic term *L*^(*k*)^, The score function is $E(L(p))=S_{\bar{\alpha }}$, where $\bar{\alpha }=\sum_{k=1}^{\#L(p)}r^{\left(k\right)}p^{\left(k\right)}/\sum_{k=1}^{\#L(p)}p^{\left(k\right)}$, The deviation degree of *L*(*p*) is given by:

$$\sigma (L(p))=(\sum_{k=1}^{\#L(p)}{\left(p^{\left(k\right)}(r^{\left(k\right)}-\bar{\alpha })\right)}^{2}{)}^{1/2}/\sum_{k=1}^{\#L(p)}p^{\left(k\right)}$$ (16)

Let *L*(*p*)_1_ and *L*(*p*)_2_ be two PLTS with score functions *E*(*L*(*p*)_1_) and *E*(*L*(*p*)_2_), and deviation degrees σ(*L*(*p*)_1_) and σ(*L*(*p*)_2_), respectively. When *E*(*L*(*p*)_1_) = *E*(*L*(*p*)_2_), if σ(*L*(*p*)_1_) < σ(*L*(*p*)_2_), then *L*(*p*)_1_ > *L*(*p*)_2_; if σ(*L*(*p*)_1_) = σ(*L*(*p*)_2_), then *L*(*p*)_1_ = *L*(*p*)_2_; if σ(*L*(*p*)_1_) > σ(*L*(*p*)_2_), then *L*(*p*)_1_ < *L*(*p*)_2_.

Example 5. A certain project has two options, Plan A and Plan B. The development team needs to assess the project success rate of two projects, and the experts have shown hesitation in their views on the risks. Experts believe that success rate for Project A is {(*s*_4_,0.25), (*s*_6_,0.75)}. Experts believe that success rate for Project B is {(*s*_4_,0.5), (*s*_6_,0.5)}. By calculating their score functions using Formula 15, the results can be obtained as follows.


$$S(L{\left(p\right)}_{A})=S_{\bar{\alpha }}=S_{\frac{4*0.25+6*0.75}{0.25+0.75}}=S_{5.5},S(L{\left(p\right)}_{B})=S_{\bar{\alpha }}=S_{\frac{4*0.5+6*0.5}{0.25+0.75}}=S_{5}.$$

## 4. Evaluation framework for urban NEVs development prospects based on IEntropy-Mo-RVIKOR

**Fig 3:**
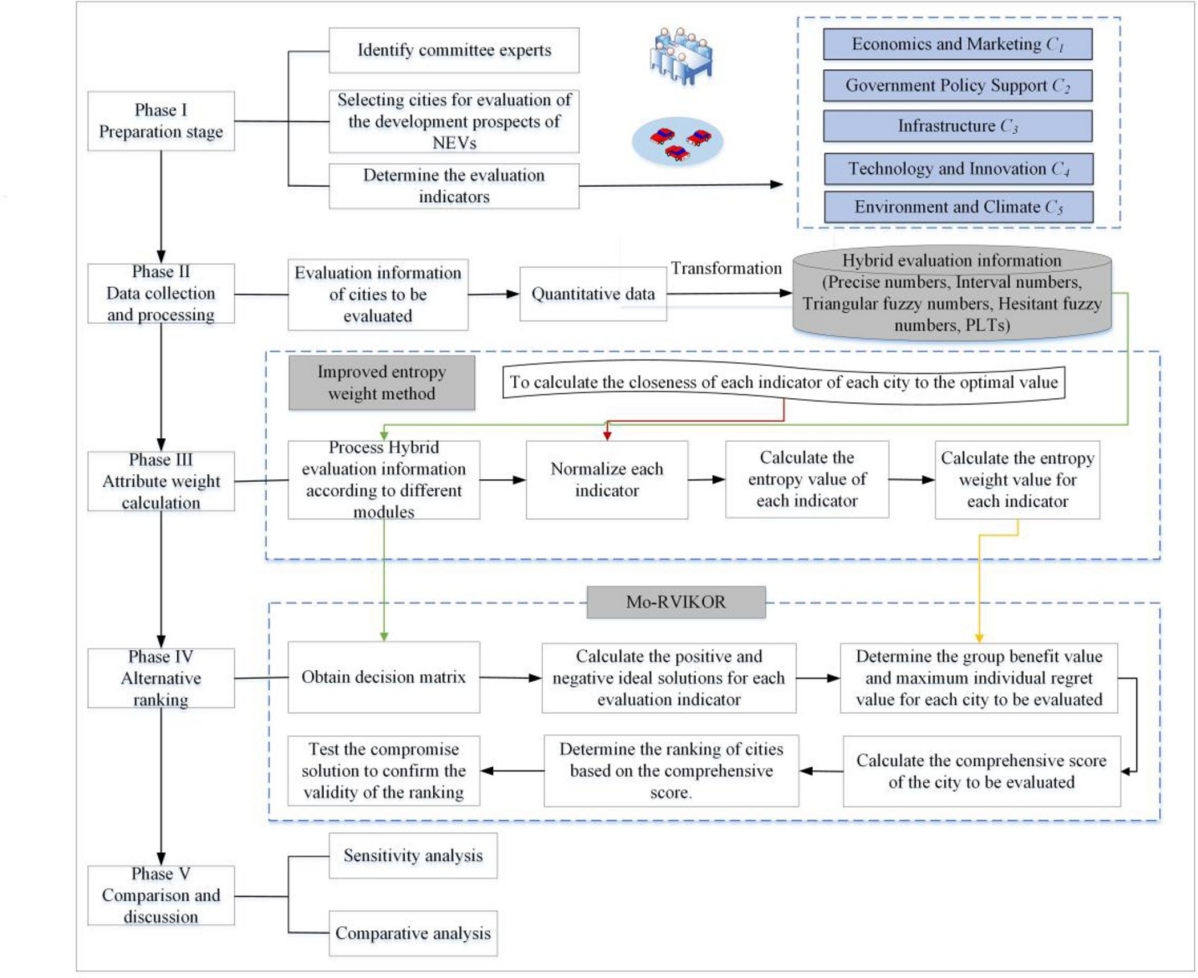
The framework for evaluating the development prospects of urban NEVs.

This section develops an evaluation framework for the development prospects of urban NEVs based on the IEntropy-Mo-RVIKOR method, according to the evaluation index system outlined in section 2.2, as shown in Fig 3. The following is a detailed introduction to the specific calculation process of this framework.

### 4.1. Evaluation information collection

Assume the cities to be evaluated are denoted as *A*_*i*_ (*i* = 1,2,⋯,*m*), and the evaluation indicators are denoted as *C*_*j*_ (*j* = 1,2,⋯,*n*). All indicators are categorized, and experts *E*_*k*_ (*k* = 1,2,⋯,*t*)evaluate the indicators based on relevant attributes and their own assessment habits, choosing from precise numbers, interval numbers, triangular fuzzy numbers, hesitant fuzzy numbers, and PLTS. Here, *x*_*ij*_ represents the evaluation information of city *A*_*i*_ for criterion *C*_*j*_. There are five modules, with the superscript *N* indicating the evaluation information is numerical, *I* indicating interval numbers, *T* indicating triangular fuzzy numbers, *F* indicating hesitant fuzzy numbers, and *P* indicating PLTS. After standardizing the evaluation information, the aggregated evaluation information matrix is as follows:

$$X={\left(x_{ij}\right)}_{m\times n}=(M_{1},M_{2},\cdots ,M_{5})=\left[\begin{array}{l} x_{11}^{N}\;\;x_{12}^{N}\;\;\cdots \;\;x_{1h}^{I}\;\;x_{1(h+1)}^{I}\;\;\cdots \;\;x_{1k}^{T}\;\;x_{1(k+1)}^{T}\;\;\cdots x_{1l}^{F}\;\;x_{1(l+1)}^{F}\;\;\cdots x_{1(n-1)}^{P}\;\;x_{1n}^{P}\\ x_{21}^{N}\;\;x_{22}^{N}\;\;\cdots \;\;x_{2h}^{I}\;\;x_{2(h+1)}^{I}\;\;\cdots \;\;x_{2k}^{T}\;\;x_{2(k+1)}^{T}\;\;\cdots x_{2l}^{F}\;\;x_{2(l+1)}^{F}\;\;\cdots x_{2(n-1)}^{P}\;\;x_{2n}^{P}\\ \;\vdots \\ x_{i1}^{N}\;\;x_{i2}^{N}\;\;\cdots \;\;x_{ih}^{I}\;\;x_{i(h+1)}^{I}\;\;\cdots \;\;x_{ik}^{T}\;\;x_{i(k+1)}^{T}\;\;\cdots \;x_{il}^{F}\;\;x_{i(l+1)}^{F}\;\;\cdots x_{i(n-1)}^{P}\;\;x_{in}^{P}\\ \;\vdots \\ x_{m1}^{N}\;\;x_{m2}^{N}\;\;\cdots \;\;x_{mh}^{I}\;\;x_{m(h+1)}^{I}\;\;\cdots \;\;x_{mk}^{T}\;\;x_{m(k+1)}^{T}\;\;\cdots x_{ml}^{F}\;\;x_{m(l+1)}^{F}\;\;\cdots x_{m(n-1)}^{P}\;\;x_{mn}^{P} \end{array}\right]$$ (17)

Where, *x*_*ij*_ represents the expert integrated numerical evaluation value of the *j*-th indicator for city *A*_*i*_.

### 4.2. Calculating the weights of evaluation indicators based on the IEntropy method

In the process of calculating using the entropy method, it is necessary to normalize the evaluation information. Considering that the evaluation information in this paper is mixed, the mixed evaluation information is processed by modules according to different modules. The processing method is as follows:

$$X^{\prime }={X^{\prime }}_{ij}=\left[\begin{array}{l} \frac{x_{11}^{N}}{{\left(x_{m1}^{N}\right)}^{*}},\;\;\frac{x_{12}^{N}}{{\left(x_{m2}^{N}\right)}^{*}}\;\;\cdots \;\;\frac{S(x_{1h}^{I})}{S{\left(x_{mh}^{I}\right)}^{*}},\;\;\frac{S(x_{1(h+1)}^{I})}{S{\left(x_{m(h+1)}^{I}\right)}^{*}}\;\;\cdots \;\;\frac{S(x_{1k}^{T})}{S{\left(x_{mk}^{T}\right)}^{*}},\;\;\frac{S(x_{1(k+1)}^{T})}{S{\left(x_{m(k+1)}^{T}\right)}^{*}}\;\;\cdots \frac{S(x_{1l}^{F})}{S{\left(x_{ml}^{F}\right)}^{*}},\;\;\frac{S(x_{1(l+1)}^{F})}{S{\left(x_{m(l+1)}^{F}\right)}^{*}}\;\;\cdots \frac{S(x_{1(n-1)}^{P})}{S{\left(x_{n(n-1)}^{P}\right)}^{*}},\;\;\frac{S(x_{1n}^{P})}{S{\left(x_{mn}^{P}\right)}^{*}}\\ \frac{x_{21}^{N}}{{\left(x_{m1}^{N}\right)}^{*}},\;\;\frac{x_{22}^{N}}{{\left(x_{m2}^{N}\right)}^{*}}\;\;\cdots \;\;\frac{S(x_{2h}^{I})}{S{\left(x_{mh}^{I}\right)}^{*}},\;\;\frac{S(x_{2(h+1)}^{I})}{S{\left(x_{m(h+1)}^{I}\right)}^{*}}\;\;\cdots \;\;\frac{S(x_{2k}^{T})}{S{\left(x_{mk}^{T}\right)}^{*}},\;\;\frac{S(x_{2(k+1)}^{T})}{S{\left(x_{m(k+1)}^{T}\right)}^{*}}\;\;\cdots \frac{S(x_{2l}^{F})}{S{\left(x_{ml}^{F}\right)}^{*}},\;\;\frac{S(x_{2(l+1)}^{F})}{S{\left(x_{m(l+1)}^{F}\right)}^{*}}\;\;\cdots \frac{S(x_{2(n-1)}^{P})}{S{\left(x_{m(n-1)}^{P}\right)}^{*}},\;\;\frac{S(x_{2n}^{P})}{S{\left(x_{mn}^{P}\right)}^{*}}\\ \;\vdots \\ \frac{x_{i1}^{N}}{{\left(x_{m1}^{N}\right)}^{*}},\;\;\frac{x_{i2}^{N}}{{\left(x_{m2}^{N}\right)}^{*}}\;\;\cdots \;\;\frac{S(x_{ih}^{I})}{S{\left(x_{mh}^{I}\right)}^{*}},\;\;\frac{S(x_{i(h+1)}^{I})}{S{\left(x_{m(h+1)}^{I}\right)}^{*}}\;\;\cdots \;\;\frac{S(x_{ik}^{T})}{S{\left(x_{mk}^{T}\right)}^{*}},\;\;\frac{S(x_{i(k+1)}^{T})}{S{\left(x_{m(k+1)}^{T}\right)}^{*}}\;\;\cdots \frac{S(x_{il}^{F})}{S{\left(x_{ml}^{F}\right)}^{*}},\;\;\frac{S(x_{i(l+1)}^{F})}{S{\left(x_{m(l+1)}^{F}\right)}^{*}}\;\;\cdots \frac{S(x_{i(n-1)}^{P})}{S{\left(x_{m(n-1)}^{P}\right)}^{*}},\;\;\frac{S(x_{in}^{P})}{S{\left(x_{mn}^{P}\right)}^{*}}\\ \;\vdots \\ \frac{x_{m1}^{N}}{{\left(x_{m1}^{N}\right)}^{*}},\;\;\frac{x_{m2}^{N}}{{\left(x_{m2}^{N}\right)}^{*}}\;\;\cdots \;\;\frac{S(x_{mh}^{I})}{S{\left(x_{mh}^{I}\right)}^{*}},\;\;\frac{S(x_{m(h+1)}^{I})}{S{\left(x_{m(h+1)}^{I}\right)}^{*}}\;\;\cdots \;\;\frac{S(x_{mk}^{T})}{S{\left(x_{mk}^{T}\right)}^{*}},\;\;\frac{S(x_{m(k+1)}^{T})}{S{\left(x_{m(k+1)}^{T}\right)}^{*}}\;\;\cdots \frac{S(x_{ml}^{F})}{S{\left(x_{ml}^{F}\right)}^{*}},\;\;\frac{S(x_{m(l+1)}^{F})}{S{\left(x_{m(l+1)}^{F}\right)}^{*}}\;\;\cdots \frac{S(x_{m(n-1)}^{P})}{S{\left(x_{m(n-1)}^{P}\right)}^{*}},\;\;\frac{S(x_{mn}^{P})}{S{\left(x_{mn}^{P}\right)}^{*}} \end{array}\right]$$ (18)

Where ()^*^ represents the maximum value of the indicator for different schemes when the indicator is an exact numerical value; *S*() represents the score function of the evaluation value of the indicator for different schemes when the indicator is an imprecise value. *S*()^*^ represents the maximum value of the score function of the evaluation value of the indicator for different schemes when the indicator is an imprecise value. *h*,*k*,*l* = 1,2,⋯,*m*. The specific calculation steps of the IEntropy method are as follows:

Step 1: Calculate the *b*_*ij*_ which is the normalized results of *B*_*ij*_.


$$b_{ij}=\frac{B_{\text{ij}}}{\sum_{i=1}^{m}B_{\text{ij}}}$$ (19)

Where, *B*_*ij*_ indicates the proximity of the *j*-th indicator of the *i*-th scheme to the optimal value. $B_{ij}={X^{\prime }}_{ij}/{\left({X^{\prime }}_{ij}\right)}^{*}$, ${\left({X^{\prime }}_{ij}\right)}^{*}$ is the maximum value of the *j*-th indicator among all schemes of *X*′.

Step 2: Calculate the entropy value of each indicator *H*_*j*_.


$$H_{j}=-K\sum_{i=1}^{m}f_{ij}\text{ln}f_{ij}(i=1,2,\ldots ,m,\;j=1,2,\ldots ,n)$$ (20)

Where $f_{ij}=b_{ij}/b_{j},b_{j}=\sum_{i=1}^{m}b_{ij},K=1/\text{ln}(m).$

Step 3: Calculate the entropy weight of each indicator *ω*_*j*_.


$$\omega_{j}=\frac{\sum_{j=1}^{n}H_{j}+1-2\times H_{j}}{\sum_{j=1}^{n}\left(\sum_{j=1}^{n}H_{j}+1-2\times H_{j}\right)}(i=1,2,\ldots ,m)$$ (21)

To demonstrate the rationality of formula (21), knowing the entropy value *H*_*j*_ of the indicator set, the values $\sum_{j=1}^{n}H_{j}$ and $\sum_{j=1}^{m}(\sum_{j=1}^{m}H_{j}+1-2H_{j})$ can be determined, since *H*_*j*_ ≥ 0 and $H_{j}\in [0,1]$, it follows that the final indicator weight ω_*j*_ decreases as *H*_*j*_ increases. Therefore, the larger the entropy value, the smaller the indicator weight. Let *C*_*p*_ and *C*_*q*_ be any two indicators in the indicator set, with entropy values *H*_*p*_ and *H*_*q*_, and corresponding weights ω_*p*_ and ω_*q*_, respectively. Comparing the indicator weights, we get:

$$\frac{\omega_{p}}{\omega_{q}}=\frac{\sum_{j=1}^{n}H_{j}+1-2\times H_{p}}{\sum_{j=1}^{n}H_{j}+1-2\times H_{q}}\;\;\;p,q=\{1,2,\cdots ,n\},\;p\neq q$$ (22)

Let ε = *H*_*p*_-*H*_*q*_, which is a small variable, then formula (22) can be transformed into:

$$\frac{\omega_{p}}{\omega_{q}}=1-\frac{2\varepsilon }{\sum_{j=1}^{n}H_{j}+1-2\times H_{q}}$$ (23)

Due to the small change in the value of *H*_*j*_, and since *H*_*j*_ ∈[0,1], we can obtain:

$$\sum_{j=1}^{n}H_{j}+1-2\times H_{q}\approx 1+(n-2)H_{q}\in [1,n-1]$$ (24)

Since ε is a small variable, we can infer that $\frac{2\varepsilon }{\sum_{j=1}^{n}H_{j}+1-2\times H_{q}}$ is an even smaller variable than ε. Consequently, the change in $\frac{\omega_{p}}{\omega_{q}}$ is also very small. Therefore, using formula (21) to calculate the entropy weight will not result in multiplicative changes.

The comparison results with the traditional entropy method are shown in Table 2. By comparing, it can be found that when the entropy value approaches 1, the traditional entropy method yields very close entropy values (0.999, 0.998, 0.997), but the weight distribution varies significantly, (0.999, 0.998, 0.997), but the weight distribution varies significantly, indicating an unreasonable distribution of indicator weights. In contrast, the IEntropy method yields indicator weights of (0.333, 0.333, 0.333), indicating a more reasonable distribution. When the entropy value approaches 0.5 and 0, the results of the IEntropy method and the traditional entropy method are basically consistent. Therefore, the IEntropy method proposed in this study is more reasonable.

**Table 2:** Comparison of calculation results between traditional entropy method and the IEntropy method.

	Entropy Value	Traditional Entropy Method	IEntropy Method
When *H*_*j*_→1	(0.999,0.998,0.997) (0.9,0.8,0.7)	(0.167,0.333,0.5) (0.168,0.333,0.5)	(0.333,0.333,0.333) (0.296,0.333,0.370)
When *H*_*j*_→0.85	(0.86,0.85,0.84) (0.88,0.85,0.82)	(0.311,0.333,0.356) (0.267,0.333,0.4)	(0.33,0.333,0.337) (0.328,0.333,0.339)
When *H*_*j*_→0.6	(0.7,0.6,0.5) (0.61,0.6,0.59)	(0.25,0.333,0.4167) (0.325,0.333,0.342)	(0.288,0.333,0.379) (0.331,0.333,0.336)
When *H*_*j*_→0.5	(0.6,0.5,0.4) (0.7,0.5,0.3)	(0.267,0.333,0.4) (0.2,0.333,0.467)	(0.289,0.333,0.378) (0.244,0.333,0.422)
When *H*_*j*_→0.4	(0.5,0.4,0.3) (0.44,0.4,0.36)	(0.278,0.3333,0.389) (0.3111,0.333,0.3556)	(0.284,0.333,0.383) (0.327,0.333,0.34)
When *H*_*j*_→0.2	(0.35,0.2,0.05) (0.21,0.2,0.19)	(0.271,0.333,0.396) (0.329,0.333,0.338)	(0.281,0.333,0.386) (0.332,0.333,0.335)
When *H*_*j*_→0	(0.003,0.002,0.001) (0.3,0.2,0.1)	(0.333,0.333,0.333) (0.292,0.333,0.375)	(0.333,0.333,0.333) (0.278,0.333,0.389)

### 4.3. Urban evaluation results and ranking based on Mo-RVIKOR

#### 4.3.1. Concept and application of modular thinking

Modular thinking is a method that simplifies analysis and decision-making by breaking down complex systems into independent modules. In this study, modular thinking is used to categorize and handle mixed types of evaluation information. Specifically, evaluation indicators are divided into different modules according to their type of information—exact numbers, interval numbers, triangular fuzzy numbers, hesitant fuzzy numbers, and probabilistic linguistic term sets (PLTS). Each module processes its internal information independently, and then the information is synthesized for decision support.

#### 4.3.2. Core Principles of the Mo-RVIKOR method

Mo-RVIKOR is an improvement on the traditional VIKOR method, specially designed to handle decision-making problems with multiple types of information. The decision-making process is implemented through the following steps:

Independent Module Processing: According to modular thinking, mixed evaluation information is allocated to the corresponding modules based on the characteristics of the indicators.

Determination of Ideal and Negative Ideal Solutions: Each module independently determines its positive (optimal) and negative (worst) ideal solutions.

Calculation of Group Utility and Individual Regret: The maximum group benefit and minimum individual regret are calculated for each alternative. These metrics help to assess the overall advantages and potential weaknesses of the options.

Comprehensive Indicator Calculation and Ranking: A comprehensive evaluation indicator, combining group utility and individual regret, is used to rank all alternatives.

#### 4.3.3. The step of the proposed method

The step of urban evaluation results and ranking method based on Mo-RVIKOR is as follows.

Step 1: Using the method described in this paper, collect expert evaluation information to construct and obtain the decision matrix ***X*** (see Eq (17)). Specifically: When the evaluation information in the decision matrix is a precise value, *x*_*ij*_ = *y*_*ij*_; When the evaluation information is an interval number, *x*_*ij*_ = ${\tilde{r}}_{ij}$; When the evaluation information is a triangular fuzzy number, *x*_*ij*_ = ${\tilde{a}}_{ij}$; When the evaluation information is a hesitant fuzzy number, *x*_*ij*_ = *h*_*ij*_; When the evaluation information is a probabilistic linguistic term set, *x*_*ij*_ = *L(P)*_*ij*_. The Mo-RVIKOR urban evaluation method, considering the interrelation among demands, is as follows.

Step 2: Identify the positive ideal solution P^*^ $=\overset{m}{\underset{i=1}{\max(x_{ij})}}$ and the negative ideal solution *P*^-^$=\overset{m}{\underset{i=1}{\min(x_{ij})}}$ for each evaluation indicator based on the decision matrix provided by the experts.

Step 3: Determine the group utility *S*_*i*_ and the maximum individual regret *R*_*i*_.


$$S_{i}=\sum_{j=1}^{n}w_{j}\frac{P^{*}-x_{ij}}{P^{*}-P^{-}}$$ (25)

$$R_{i}=\max_{j}w_{j}\frac{P^{*}-x_{ij}}{P^{*}-P^{-}}$$ (26)

Where *w*_*j*_ (*j* = 1,2,…*n*)are the indicator weights obtained using the IEntropy weight method.

Step 4: Calculate the comprehensive indicator *Q*_*i*_ for the cities being evaluated.


$$Q_{i}=v\frac{S_{i}-S^{-}}{S^{*}-S^{-}}+(1-v)\frac{R_{i}-R^{-}}{R^{*}-R^{-}}$$ (27)

Where $S^{*}=\underset{i}{\max}\left\{S_{i}\right\}$, $S^{-}=\underset{i}{\min}\left\{S_{i}\right\}$; $R^{*}=\underset{i}{\max}\left\{R_{i}\right\}$, $R^{-}=\underset{i}{\min}\left\{R_{i}\right\}$. Here, *v* is the decision-making coefficient: when *v*>0.5, the decision-maker places more emphasis on group utility; when *v* = 0.5, the decision-maker’s preference is neutral; and when *v*<0.5, the decision-maker places more emphasis on the maximum individual regret.

Step 5: Sort the results in ascending order based on the comprehensive indicator *Q*_*i*_.

Step 6: Test the compromise solution. Let *A*^(1)^ be the compromise solution, which must satisfy both of the following conditions. Here, *A*^(1)^ refers to the candidate solution ranked first based on the comprehensive indicator *Q*_*i*_.

Condition 1: Acceptable reliability: $Q\left(A^{\left(2\right)}-A^{\left(1\right)}\right)\geq DQ,DQ=1/(m-1).$

Condition 2: The solution *A*^(1)^ should still rank first when evaluated by both group utility *S*_*i*_ and maximum individual regret *R*_*i*_.

If the above conditions are not simultaneously satisfied, the compromise solution is determined as follows:

1. If Condition 2 is not satisfied, both *A*^(1)^ and *A*^(2)^ are considered compromise solutions.1/(m-1)
2. If Condition 1 is not satisfied, *A*^(1)^, *A*^(2)^,…, *A*^(*x*)^ are considered optimal compromise solutions, where *x* is determined by the largest *x* satisfying the inequality *Q*(*A*^(*x*)^-*A*^(1)^)<1/(*m*-1).

## 5. Case study

### 5.1. Evaluation of urban NEVs development prospects

In this part, we apply the developed indicator system and the IEntropy-Mo-RVIKOR method to evaluate the development prospects of NEVs in ten Chinese cities.

Based on the overview of the study area in Section 2.1, the ten cities to be evaluated in this case study are labeled as *A*_*1*_ to *A*_*10*_. Additionally, using the evaluation indicator system constructed in Section 2.3, the five evaluation indicators are *C* = {*C*_*1*_: Economics and Marketing, *C*_*2*_: Government Policy Support, *C*_*3*_: Social and Infrastructure, *C*_*4*_: Technology and Innovation, *C*_*5*_: Environment and Climate }. In evaluating the ten cities for our study, we engaged five experts from distinct domains—urban planning, renewable energy, automotive engineering, environmental policy, and economic impact assessment. This panel size was intentionally chosen to balance diversity of insights and efficiency in decision-making. Research supports smaller expert groups for their focused discussions and efficient consensus-building, particularly effective in complex evaluations like ours [38, 39]. Each expert’s significant experience ensured comprehensive coverage of all critical aspects of electric vehicle infrastructure, justifying our selection for robust decision-making processes.

1. Expert *E*_*1*_ from the economic field evaluated *C*_*1*_ using precise numbers.
2. Expert *E*_*2*_ from government departments evaluated *C*_*2*_ using interval numbers.
3. Expert *E*_*3*_ from the field of sociology evaluated *C*_*3*_ using triangular fuzzy numbers.
4. Expert *E*_*4*_ from automotive engineering technology evaluated *C*_*4*_ using hesitant fuzzy numbers.
5. Expert *E*_*5*_ from the environmental science community evaluated *C*_*5*_ using PLTS.

The evaluation information obtained is shown in Table 3.

**Table 3:** Evaluation information of each attribute for alternative solutions.

City	*C*_*1*_	*C*_*2*_	*C*_*3*_	*C*_*4*_	*C*_*5*_
*A*_*1*_	40	[6,7]	{1,2,3}	H{0.1,0.2,0.4}	{(*s*_1_,0.7),(*s*_2_,0.3)}
*A*_*2*_	133	[4,6]	{5,8,9}	H{0.8,0.9}	{(*s*_4_,0.5),(*s*_5_,0.5)}
*A*_*3*_	73	[3,5]	{3,5,6}	H{0.4,0.5,0.6}	{(*s*_3_,0.3),(*s*_4_,0.5)}
*A*_*4*_	69	[2,4]	{4,5,6}	H{0.2,0.4}	{(*s*_3_,0.8),(*s*_4_,0.2)}
*A*_*5*_	106	[4,5]	{2,4,5}	H{0.4,0.5,0.6}	{(*s*_4_,0.4),(*s*_5_,0.4)}
*A*_*6*_	161	[7,8]	{7,8,9}	H{0.6,0.8}	{(*s*_7_,0.2),(*s*_8_,0.8)}
*A*_*7*_	87	[5,6]	{4,5,7}	H{0.3,0.4,0.5}	{(*s*_5_,0.3),(*s*_6_,0.7)}
*A*_*8*_	110	[5,6]	{4,5,6}	H{0.5,0.6}	{(*s*_5_,0.2),(*s*_7_,0.8)}
*A*_*9*_	92	[3,4]	{6,7,8}	H{0.5,0.6}	{(*s*_4_,0.5),(*s*_6_,0.5)}
*A*_*10*_	128	[7,9]	{6,7,9}	H{0.5,0.7}	{(*s*_6_,0.2),(*s*_8_,0.8)}

The information in Table 3 is standardized, and the final results are shown in Table 4.

**Table 4:** Standardized evaluation information of each attribute for alternative solutions.

City	*C*_*1*_	*C*_*2*_	*C*_*3*_	*C*_*4*_	*C*_*5*_
*A*_*1*_	0.04	[6,7]	{1,2,3}	H{0.1,0.2,0.4}	{(*s*_1_,0.7),(*s*_2_,0.3)}
*A*_*2*_	0.133	[4,6]	{5,8,9}	H{0.8,0.85,0.9}	{(*s*_4_,0.5),(*s*_5_,0.5)}
*A*_*3*_	0.073	[3,5]	{3,5,6}	H{0.4,0.5,0.8}	{(*s*_3_,0.375),(*s*_4_,0.625)}
*A*_*4*_	0.069	[2,4]	{4,5,6}	H{0.2,0.3,0.4}	{(*s*_3_,0.8),(*s*_4_,0.2)}
*A*_*5*_	0.106	[4,5]	{2,4,5}	H{0.4,0.5,0.6}	{(*s*_4_,0.5),(*s*_5_,0.5)}
*A*_*6*_	0.161	[7,8]	{7,8,9}	H{0.6,0.7,0.8}	{(*s*_7_,0.2),(*s*_8_,0.8)}
*A*_*7*_	0.087	[5,6]	{4,5,7}	H{0.3,0.4,0.5}	{(*s*_5_,0.3),(*s*_6_,0.7)}
*A*_*8*_	0.11	[5,6]	{4,5,6}	H{0.5,0.55,0.6}	{(*s*_5_,0.2),(*s*_7_,0.8)}
*A*_*9*_	0.092	[3,4]	{6,7,8}	H{0.5,0.55,0.6}	{(*s*_4_,0.5),(*s*_6_,0.5)}
*A*_*10*_	0.128	[7,9]	{6,7,9}	H{0.5,0.6,0.7}	{(*s*_6_,0.2),(*s*_8_,0.8)}

The score functions of the evaluation information in Table 3 are calculated and normalized, and the results are shown in Table 5. The *b*_*ij*_ values in formula (19) are calculated, and the results are shown in Table 6. The entropy values *H*_*j*_ for each indicator are calculated according to formula (20): *H*_*1*_ = 0.285, *H*_*2*_ = 0.269, *H*_*3*_ = 0.262, *H*_*4*_ = 0.2889, *H*_*5*_ = 0.284.

**Table 5:** Normalized evaluation information based on score functions.

City	*C*_*1*_	*C*_*2*_	*C*_*3*_	*C*_*4*_	*C*_*5*_
*A*_*1*_	0.251	0.8125	0.325	0.275	0.167
*A*_*2*_	0.827	0.625	0.95	1	0.577
*A*_*3*_	0.452	0.5	0.6	0.588	0.372
*A*_*4*_	0.428	0.375	0.625	0.353	0.41
*A*_*5*_	0.661	0.5625	0.475	0.588	0.462
*A*_*6*_	1	0.9375	1	0.824	1
*A*_*7*_	0.542	0.6875	0.65	0.471	0.731
*A*_*8*_	0.685	0.6875	0.625	0.647	0.846
*A*_*9*_	0.572	0.4375	0.875	0.647	0.641
*A*_*10*_	0.793	1	0.9	0.706	0.949

**Table 6:** Calculation results of *b*_*ij*_.

City	*C*_*1*_	*C*_*2*_	*C*_*3*_	*C*_*4*_	*C*_*5*_
*A*_*1*_	0.040	0.123	0.046	0.045	0.027
*A*_*2*_	0.133	0.094	0.135	0.164	0.094
*A*_*3*_	0.073	0.075	0.085	0.096	0.060
*A*_*4*_	0.069	0.057	0.089	0.058	0.067
*A*_*5*_	0.106	0.085	0.068	0.096	0.075
*A*_*6*_	0.161	0.142	0.142	0.135	0.162
*A*_*7*_	0.087	0.104	0.093	0.077	0.119
*A*_*8*_	0.110	0.104	0.089	0.106	0.137
*A*_*9*_	0.092	0.066	0.125	0.106	0.104
*A*_*10*_	0.128	0.151	0.128	0.116	0.154

The weights of each indicator are calculated according to formula (21): ω_1_ = 0.284, ω_1_ = 0.244, ω_1_ = 0.205, ω_1_ = 0.155, ω_1_ = 0.112.

Using the Mo-RVIKOR method and formulas (26), (27), and (28), the values of *S*_*i*_,*R*_*i*_, and *Q*_*i*_ are calculated and sorted in ascending order. When *v* = 0.5, the results are shown in the Table 7:

**Table 7:** Comprehensive ranking evaluation based on Mo-RVIKOR (*v* = 0.5).

City	*S*_*i*_	Rank	*R*_*i*_	Rank	*Q*_*i*_	Rank
*A*_*1*_	0.837	10	0.284	10	1	10
*A*_*2*_	0.299	3	0.161	4	0.43	3
*A*_*3*_	0.717	8	0.215	7	0.789	8
*A*_*4*_	0.817	9	0.269	9	0.958	9
*A*_*5*_	0.637	7	0.188	6	0.686	6
*A*_*6*_	0.065	1	0.038	1	0.038	1
*A*_*7*_	0.564	5	0.174	5	0.613	5
*A*_*8*_	0.464	4	0.135	3	0.473	4
*A*_*9*_	0.566	6	0.242	8	0.753	7
*A*_*10*_	0.148	2	0.078	2	0.171	2

Testing the Compromise Solution:

Condition 1: *DQ* = 1/(10–1) = 0.111, *Q*(*A*^(2)^ -*A*^(1)^) = 0.171–0.038 = 0.133>*DQ*.

Condition 2: Satisfied.

Therefore, *A*_*6*_ is the city with the greatest potential for NEVs development among all cities. The final ranking of the cities is as follows: $A_{6}>A_{10}>A_{2}>A_{8}>A_{7}>A_{5}>A_{9}>A_{3}>A_{4}>A_{1}$.

### 5.2. Sensitivity analysis

**Fig 4:**
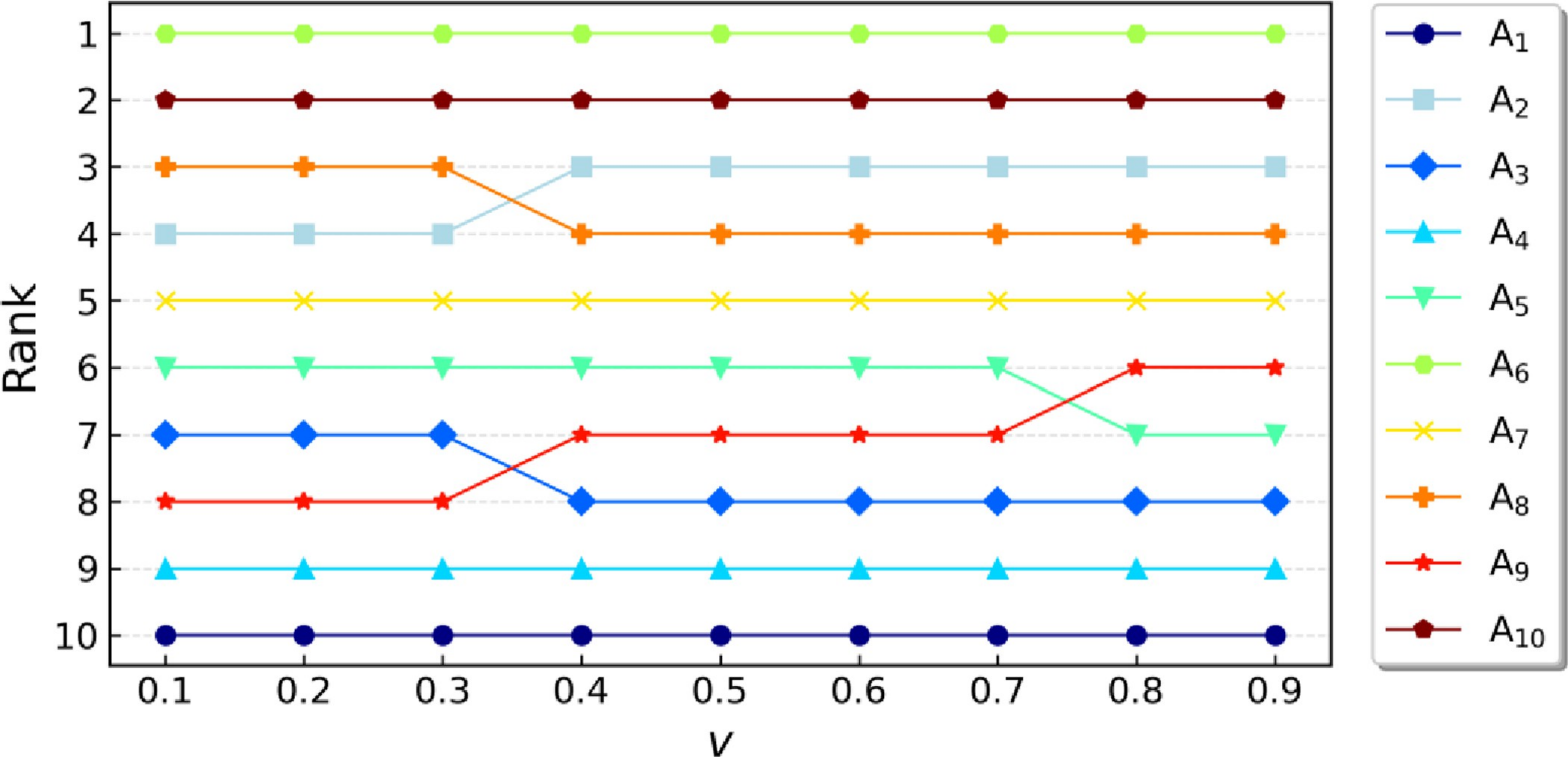
Ranking of the ten cities with different *v* values.

**Fig 5:**
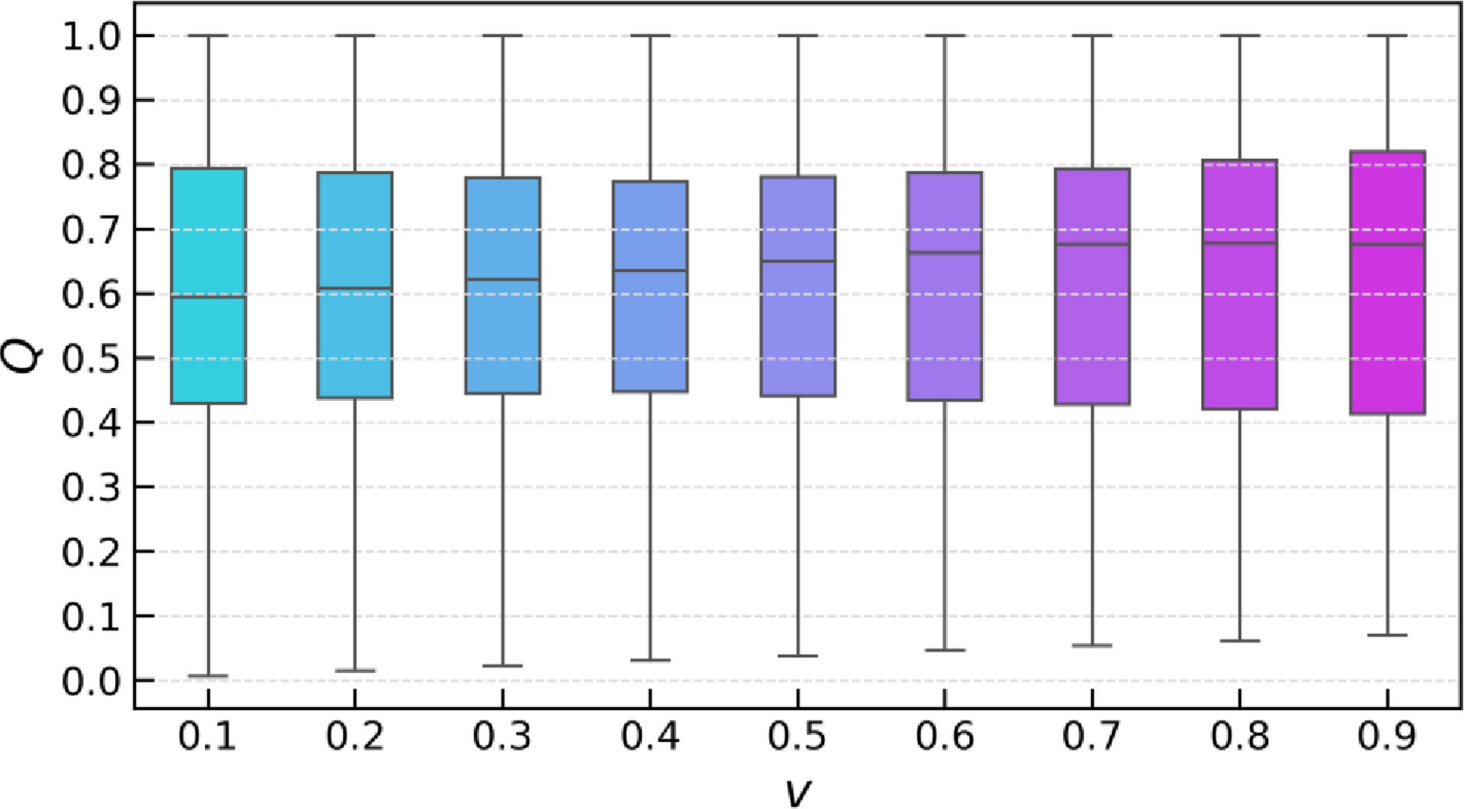
Statistical distribution of *Q* values with different *v* values.

In this part, a sensitivity analysis of the model is conducted. The weight of the decision strategy *v* was initially set to 0.5 during the implementation phase. In this study, the weight will fluctuate between 0.1 and 0.9 at intervals of 0.1. Table 8 shows the *Q* values and the rankings of the alternative solutions based on these values, highlighting the score differences between the alternative solutions for each decision strategy weight studied. Fig 4 shows the ranking changes of decision objects *A*_*1*_ to *A*_*10*_ under different values of the parameter *v*. Fig 5 shows the distribution of the decision results (*Q* values) of the IEntropy -Mo-RVIKOR model under different *v* values.

**Table 8:** Sensitivity analysis results depending on the decision strategy weight.

*v*	*Q* Values										Rank
	*A*_*1*_	*A*_*2*_	*A*_*3*_	*A*_*4*_	*A*_*5*_	*A*_*6*_	*A*_*7*_	*A*_*8*_	*A*_*9*_	*A*_*10*_	
*v* = 0.1	1.000	0.487	0.734	0.943	0.626	0.006	0.564	0.409	0.814	0.166	$A_{6}>A_{10}>A_{2}>A_{8}>A_{7}>A_{5}>A_{3}>A_{9}>A_{4}>A_{1}$
*v* = 0.2	1.000	0.473	0.748	0.947	0.641	0.014	0.576	0.425	0.799	0.167	$A_{6}>A_{10}>A_{2}>A_{8}>A_{7}>A_{5}>A_{3}>A_{9}>A_{4}>A_{1}$
*v* = 0.3	1.000	0.458	0.761	0.950	0.656	0.022	0.588	0.441	0.784	0.168	$A_{6}>A_{10}>A_{2}>A_{8}>A_{7}>A_{5}>A_{3}>A_{9}>A_{4}>A_{1}$
*v* = 0.4	1.000	0.444	0.775	0.954	0.671	0.030	0.600	0.457	0.768	0.170	$A_{6}>A_{10}>A_{8}>A_{2}>A_{7}>A_{5}>A_{9}>A_{3}>A_{4}>A_{1}$
*v* = 0.5	1.000	0.430	0.789	0.958	0.686	0.038	0.613	0.473	0.753	0.171	$A_{6}>A_{10}>A_{2}>A_{8}>A_{7}>A_{5}>A_{9}>A_{3}>A_{4}>A_{1}$
*v* = 0.6	1.000	0.415	0.802	0.962	0.701	0.046	0.625	0.490	0.738	0.172	$A_{6}>A_{10}>A_{2}>A_{8}>A_{7}>A_{5}>A_{9}>A_{3}>A_{4}>A_{1}$
*v* = 0.7	1.000	0.401	0.816	0.966	0.716	0.054	0.637	0.506	0.723	0.173	$A_{6}>A_{10}>A_{2}>A_{8}>A_{7}>A_{5}>A_{9}>A_{3}>A_{4}>A_{1}$
*v* = 0.8	1.000	0.386	0.830	0.969	0.731	0.061	0.649	0.522	0.707	0.175	$A_{6}>A_{10}>A_{2}>A_{8}>A_{7}>A_{9}>A_{5}>A_{3}>A_{4}>A_{1}$
*v* = 0.9	1.000	0.372	0.843	0.973	0.746	0.069	0.662	0.538	0.692	0.176	$A_{6}>A_{10}>A_{2}>A_{8}>A_{7}>A_{9}>A_{5}>A_{3}>A_{4}>A_{1}$

As shown in Fig 4, certain decision objects have relatively stable rankings, such as *A*_*1*_ and *A*_*6*_, indicating that they are not sensitive to changes in the parameter *v*. In contrast, other decision objects like *A*_*2*_, *A*_*3*_, *A*_*5*_, *A*_*8*_, and *A*_*9*_ exhibit noticeable upward or downward trends as the *v* value increases. This reveals that the importance of these objects in the decision-making process is closely related to the *v* value, which in turn affects their relative priority. Specifically, *A*_*3*_’s ranking continuously rises with an increasing *v* value, suggesting that in a decision-making environment with a higher tolerance for risk, *A*_*3*_ becomes a more attractive choice. Conversely, *A*_*9*_’s ranking shows a downward trend as the *v* value increases, which may indicate that its priority would increase under conditions favoring group utility. Additionally, the rankings of *A*_*5*_ and *A*_*8*_ show significant changes at certain specific points of *v*, which may point out that decision-makers should re-evaluate the importance of these decision objects at these points.

As observed in Fig 5, as the *v* value increases, the median of the decision results steadily rises, and the interquartile range gradually narrows. This indicates that the model has good adaptability and consistency in response to changes in the parameter *v*. This phenomenon shows that the model can robustly provide decision-making suggestions under different decision preference conditions, and decision-makers can formulate strategies based on the model results. From a statistical perspective, the narrowing of the interquartile range suggests that as the *v* value increases, the consistency of the decision results is enhanced, reducing the potential differences that might occur under varying decision preferences, ensuring the stability of the decision recommendations. Moreover, the general upward trend of the box plots also reveals the model’s effectiveness in capturing and distinguishing the dynamic behavior of different decision objects under changing decision preferences. Whether the decision environment favors group utility or maximum individual regret, the IEntropy -Mo-RVIKOR model can provide consistent and reliable results.

### 5.3. Comparative analysis

#### 5.3.1. Weight comparison

**Fig 6:**
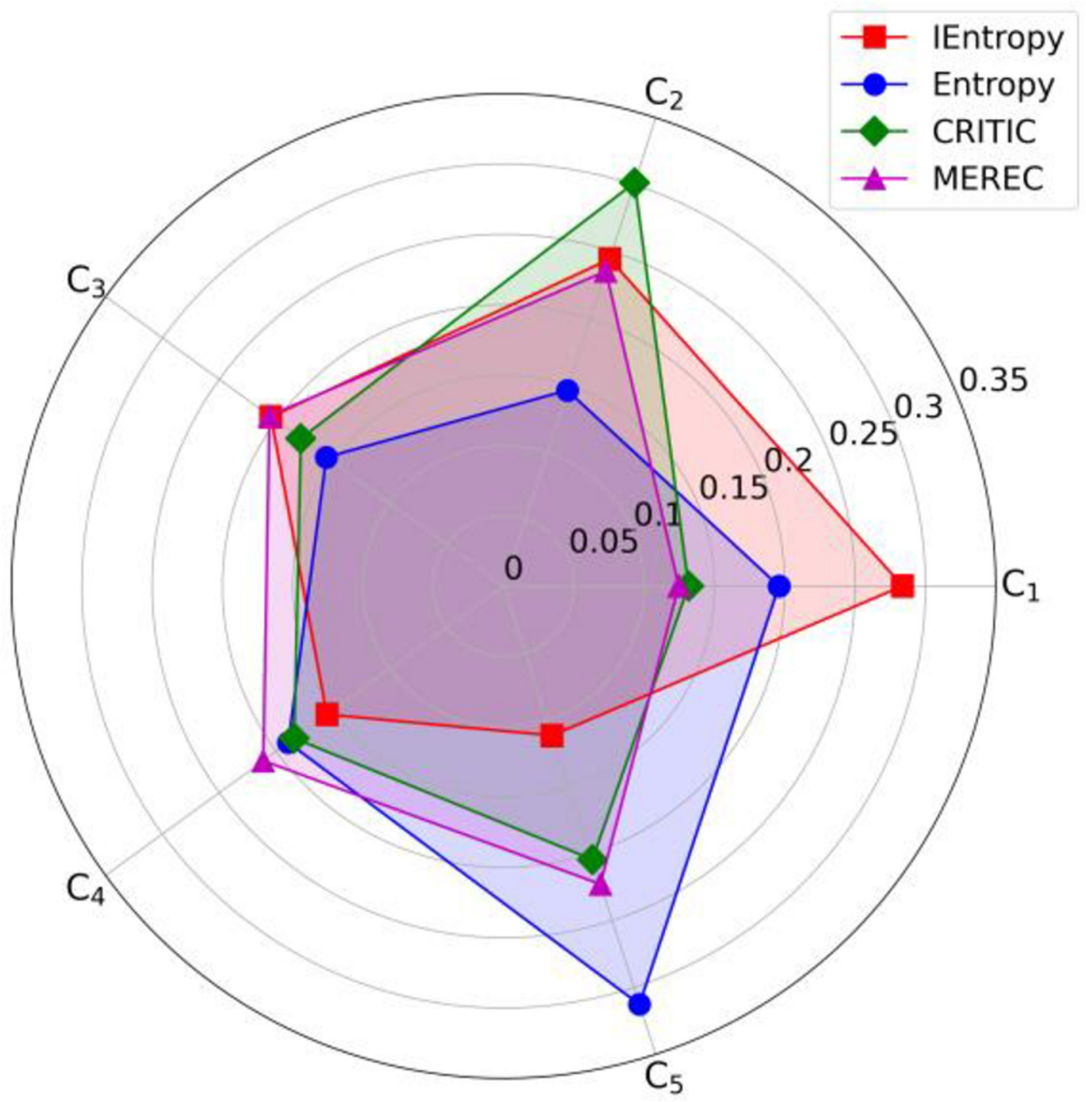
Comparison of weight calculation results using four objective weighting methods.

Fig 6 compares the weight calculation results using four objective weighting methods: the entropy method [40], the CRITIC method [41], the MEREC method [42], and the IEntropy method used in this paper. Fig 6 reveals the differences in weight allocation among the various methods. The IEntropy method emphasizes the importance of Economy and Market and Government Policy Support, demonstrating its high sensitivity to factors influencing the new energy vehicle market development. This method promotes a more reasonable and comprehensive weight distribution by considering the interrelationships and information redundancy among indicators, reflecting a higher level of scientific accuracy and practical relevance. Compared to the traditional entropy method, which focuses more on Environment and Climate, the CRITIC method highlights the role of Government Policy, while the MEREC method provides a more balanced weight distribution. The IEntropy method balances the various influencing factors, ensuring the comprehensiveness and accuracy of the evaluation results. In summary, the IEntropy method shows better comprehensiveness and adaptability in evaluating the development prospects of new energy vehicles, aligning well with market and policy-driven actual needs, making it an effective and scientific weight calculation method.

#### 5.3.2. Comparison of ranking results

To verify the feasibility and effectiveness of the method proposed in this section, we compared the IEntropy-Mo-RVIKOR method with several other methods, including Entropy-VIKOR [40], CRITIC-VIKOR [41], MEREC-VIKOR [42], IEntropy-TOPSIS [43], IEntropy-COPRAS [44], IEntropy-COCOSO [45], and IEntropy-EDAS [46], in the evaluation of urban new energy vehicle development prospects.

**Fig 7:**
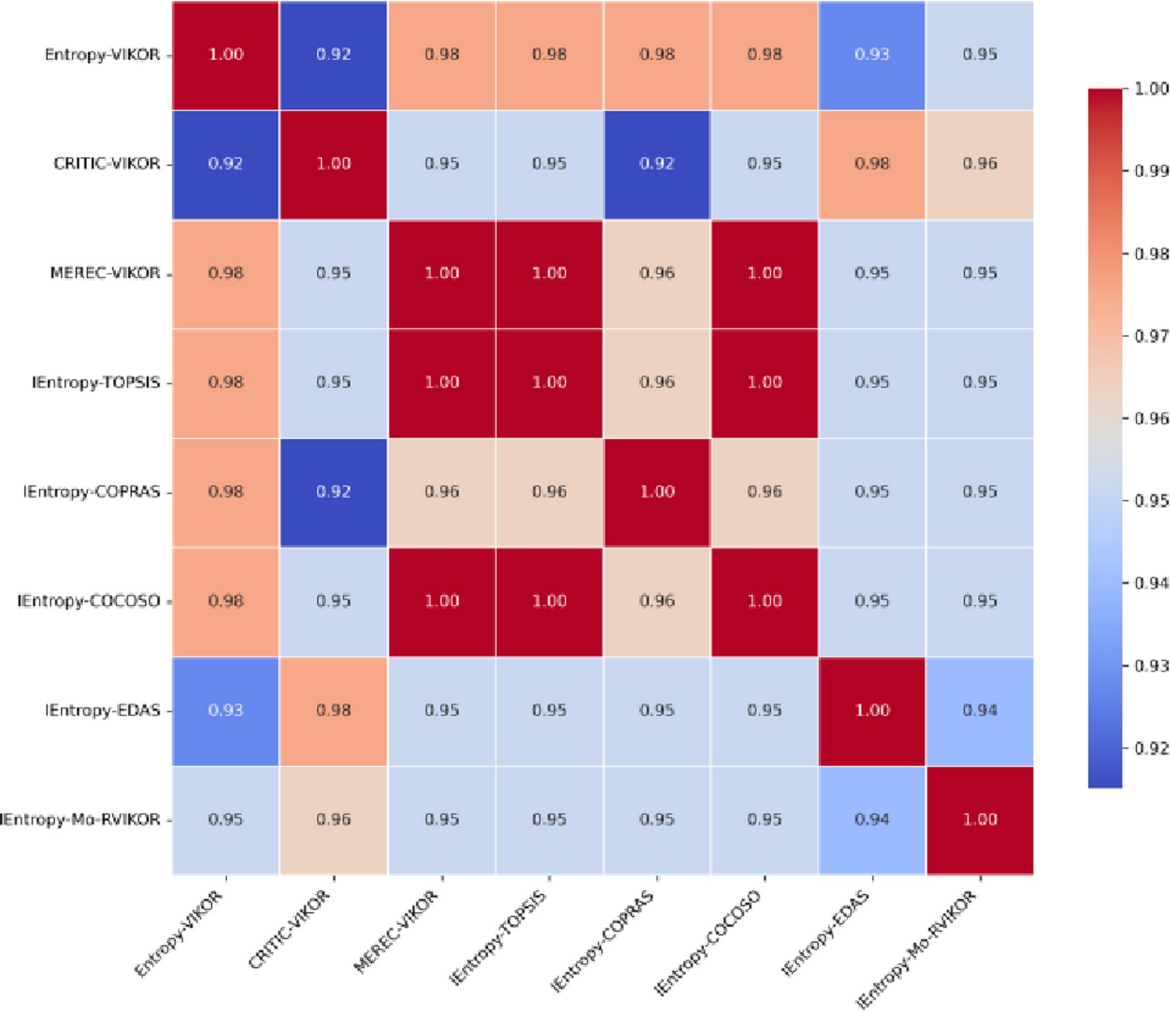
Pearson correlation coefficient of the ranking results.

As shown in Fig 7, there is a high correlation between the ranking results of different multi-attribute decision-making methods. The correlation coefficients between the IEntropy-Mo-RVIKOR method and other methods are generally above 0.90. This result clearly demonstrates the stability and reliability of the IEntropy-Mo-RVIKOR method across various decision-making scenarios. The heatmap reveals that the IEntropy-Mo-RVIKOR method not only shows a high degree of similarity with entropy-based methods such as IEntropy-TOPSIS, IEntropy-COPRAS, IEntropy-COCOSO, and IEntropy-EDAS, but also displays consistency with other VIKOR variants like Entropy-VIKOR, CRITIC-VIKOR, and MEREC-VIKOR. This indicates that the IEntropy-Mo-RVIKOR method effectively captures the essence of decision-making problems and provides decision recommendations similar to those of other well-established methods. The visual comparison through the heatmap confirms that the IEntropy-Mo-RVIKOR model not only performs well in a single decision-making environment but also demonstrates stability across multiple models, showcasing its superiority in handling real-world decision problems. Additionally, the high correlation suggests that the model can serve as a reliable tool for diverse decision-making environments.

Table 9 displays the comparative rankings of ten Chinese cities in terms of their new energy vehicle development prospects, evaluated using multiple decision-making methods. In these methods, *A*_*1*_ and *A*_*3*_ are usually ranked last or second to last, while *A*_*6*_ and *A*_*10*_ consistently hold the top two positions across all decision-making methods. This ranking consistency highlights the leading positions of Shanghai and Guangzhou in the development of the new energy vehicle industry, reflecting the consistency and reliability of multi-attribute decision-making methods in identifying and evaluating differences and specific advantages among cities. Specifically, the IEntropy-Mo-RVIKOR method improves the accuracy and robustness of the evaluation by combining the IEntropy method with the multi-indicators decision-making process (Mo-RVIKOR). This method effectively handles uncertainty and ambiguity, providing accurate and comprehensive decision support for evaluating urban NEVs development.

**Table 9:** Comparative ranking of ten Chinese cities in NEVs development prospects by multiple MADM methods.

Method	*A*_*1*_	*A*_*2*_	*A*_*3*_	*A*_*4*_	*A*_*5*_	*A*_*6*_	*A*_*7*_	*A*_*8*_	*A*_*9*_	*A*_*10*_
IEntropy-Mo-RVIKOR	10	3	8	9	6	1	5	4	7	2
Entropy-VIKOR	10	4	8	9	7	1	6	3	5	2
CRITIC-VIKOR	8	3	9	10	6	1	5	4	7	2
MEREC-VIKOR	9	3	8	10	7	1	6	4	5	2
IEntropy-TOPSIS	9	3	8	10	7	1	6	4	5	2
IEntropy-COPRAS	10	3	9	8	7	1	6	4	5	2
IEntropy-COCOSO	9	3	8	10	7	1	6	4	5	2
IEntropy-EDAS	8	3	10	9	7	1	5	4	6	2

## 6. Discussion

Based on the results of the eight evaluation methods listed in Table 9, the K-means [47] clustering algorithm groups the ten major cities into three clusters: First Group: Shanghai, Guangzhou, Beijing, Chongqing; Second Group: Chengdu, Wuhan, Xi’an; Third Group: Tianjin, Harbin, Zhengzhou.

Overall, the evaluation rankings of the ten cities are significantly consistent with the "economic market" indicators. This indicator reflects factors such as GDP, income levels, population size, and market penetration rates, which determine consumer consumption concepts and habits. Although NEVs have new features and trends, they are still a type of commodity in the market, influenced by income levels, supply and demand prices, and market environment.

### 6.1. Discussion on the first group of cities

The evaluation results show that Shanghai, Guangzhou, Beijing, and Chongqing are all ranked in the top four. Except for the Entropy-VIKOR method, the rankings are consistent across other methods. In 2022, these cities were among the top in China’s city GDP rankings, and their permanent populations were also among the top five. These four cities are located in different geographical locations in China, with higher levels of economic development and population size, making consumers more receptive to emerging things, which explains their leading position in the development of NEVs.

### 6.2. Discussion on the second group of cities

Chengdu, Wuhan, and Xi’an show differences in GDP and population size. Chengdu and Wuhan have similar GDPs, while Xi’an’s GDP is relatively lower. In terms of permanent population, Chengdu exceeds 21 million, while Wuhan and Xi’an have about 13 million. Geographically, Chengdu and Wuhan are located in the south, and Xi’an is in the north. The geographical environment of Chengdu and Wuhan is more suitable for the use of NEVs. Additionally, Xi’an’s relatively conservative consumption concept also affects the development of NEVs.

### 6.3. Discussion on the third group of cities

Geographically, Harbin, the capital of Heilongjiang Province and China’s northernmost provincial capital, faces unique challenges in NEVs adoption. Harbin’s economic indicators, including GDP, income levels, and population size, rank lowest among the evaluated cities. Its per capita income is only about one-third of that in Beijing and Shanghai, and the city suffers from significant population outflow and aging demographics, which limit consumer demand. Additionally, cold winters impact NEVs performance, and the city’s limited infrastructure for NEVs, such as charging stations, further restricts adoption.

Tianjin, despite a relatively high GDP, missed growth in emerging industries like electronics and e-commerce, remaining reliant on traditional sectors such as metallurgy and port logistics. The city’s proximity to Beijing also causes a “siphoning effect” on talent and capital, resulting in lower economic vitality and household incomes, which hampers the local NEVs market.

As the capital of Henan Province, Zhengzhou has a predominantly labor-intensive economy. Unlike cities with vehicle license restrictions that promote NEVs adoption, Zhengzhou allows free choice between conventional and new energy vehicles. Additionally, Zhengzhou’s conservative consumer culture limits NEVs demand, similar to patterns seen in other northern cities.In summary, the level of economic development and population size of a city significantly affect the development of NEVs. The higher the economic level and the larger the population size, the better the overall development of NEVs.

## 7. Conclusions and recommendations

### 7.1. Conclusions

This study constructed a comprehensive indicator system including economy and market, government policy support, infrastructure, technology and innovation, and environment and climate. It developed a novel evaluation framework based on the IEntropy-Mo-RVIKOR method to evaluate the development prospects of NEVs in Chinese cities. This method integrates various types of information, providing a means of quantitative analysis to handle mixed evaluation information in the decision matrix. The study results show that Shanghai and Guangzhou demonstrate significant development advantages across all evaluation dimensions, while Harbin and Tianjin perform relatively weakly. Sensitivity analysis and comparative analysis validate the stability and reliability of the IEntropy-Mo-RVIKOR method, providing a scientific basis for evaluating NEVs development prospects.

In summary, this paper focuses on evaluating the development prospects of urban NEVs in China and proposes a novel MADM model. The potential contributions of this paper are as follows:

Constructing a new indicator system for evaluating the development prospects of urban NEVs, which can accommodate various forms of information representation.

Proposing the Mo-RVIKOR ranking method based on modular thinking to rank major cities, reducing information loss and calculation complexity associated with hybrid evaluation information.

Introducing an IEntropy weight method to determine indicator weights, enhancing the scientific nature of weighting in evaluating urban NEVs development prospects.

Providing policymakers with an evaluation framework for urban NEVs development prospects, with the research results offering scientific support for future policy adjustments and resource allocation in various cities.

### 7.2. Policy recommendations

In recent years, the rapid development of China’s NEVs market has been driven by urban economic growth, government financial policies (subsidies and tax reductions), and NEVs enterprises breaking traditional fuel vehicle patent barriers. To further promote the adoption of NEVs in major Chinese cities, the following policy recommendations are proposed:

1. Accelerate the Establishment of NEVs Standards. The rapid development of the NEVs market requires the timely formulation of relevant standards, especially in production, replacement, recycling, and safety. Establishing standards is fundamental to the healthy and orderly development of the industry.
2. Continue Government Support Policies. Government policies have played a crucial role in the development of NEVs in China. It is essential to continue supporting the NEVs industry through subsidies, tax, financial, and insurance policies.
3. Improve Urban Infrastructure. Urban infrastructure, especially charging facilities, is critical to the development of NEVs. The government should ensure adequate supply and systematic planning, including grid layout, fire safety, and service areas.
4. Promote R&D through Differential Subsidies. Shift from universal subsidies to support for breakthrough technologies and innovative products. This approach will enhance R&D capabilities and upgrade the NEVs industry chain.
5. Strengthen Market Regulation. Strengthen market regulation to protect consumer rights and address issues such as battery warranties and replacement. Effective regulation ensures fair market behavior and promotes a smooth transition from fuel vehicles to NEVs.
6. Support NEVs Adoption in Developing Regions. In regions with slower NEVs adoption, such as Harbin and Zhengzhou, NEVs growth relies on gradual market acceptance and information dissemination. These areas tend to have conservative consumer attitudes, requiring time for market recognition. Continued technological advancements, increased competition among manufacturers, and the deepening influence of national policies will foster market growth. As NEVs penetration rises across China, it will stimulate demand in these regions. Local governments should strengthen infrastructure development and implement tailored policies to address specific regional needs.

Based on the above policy recommendations, the government and relevant decision-making bodies can more effectively promote the healthy development of the NEVs industry, contributing to the realization of national long-term environmental and energy strategic goals. Future work should further optimize the IEntropy-Mo-RVIKOR method, explore its applicability to other decision-making problems, and continuously improve its theoretical and methodological foundation in practice to provide more comprehensive policy support and decision-making references.

### 7.3. Research limitations

This study, while providing initial insights into the Mo-RVIKOR hybrid multi-attribute decision-making method for the evaluation of electric vehicles in China, has several limitations that need to be acknowledged to better understand its strengths and weaknesses:

Indicator Selection: The choice of indicators might not cover all relevant aspects that influence the performance and acceptance of electric vehicles. The selection process could be biased by the availability of data and the subjective preferences of researchers, which may not comprehensively reflect the multifaceted nature of electric vehicle evaluation.

Expert Participation: The study relied on a limited number of experts for evaluating the suitability and effectiveness of the Mo-RVIKOR method. A larger, more diverse panel of experts might provide a broader range of insights and contribute to a more robust validation of the decision-making framework.

Methodological Constraints: The Mo-RVIKOR method, while effective in handling complex decision-making scenarios, may not fully capture the dynamics of rapidly evolving industries like electric vehicles. The method’s reliance on static data inputs and its less dynamic nature might limit its applicability in environments where real-time data and continuous updates are crucial.

### 7.4. Research prospect

Building on the current research, there are several avenues for future investigation that could enhance the effectiveness and applicability of the Mo-RVIKOR method:

1. Optimization and Innovation in Data Processing:
  (1) Data Pre-processing Techniques: Developing more efficient data cleaning and preprocessing techniques to improve data quality and ensure the accuracy of analysis results.
  (2) Advanced Statistical Techniques: Integrating advanced statistical methods and machine learning algorithms to more accurately process and interpret complex datasets.
  (3) Real-time Data Processing: Incorporating real-time data processing frameworks to address the dynamic nature of data in the electric vehicle industry and achieve more flexible and responsive decision-support systems.
2. Global Evaluation of Electric Vehicles:
  (1) Transnational Data Collection: Collaborating with international organizations to collect and analyze electric vehicle usage data from different countries and regions.
  (2) Comparative Studies: Conducting comparative analyses across countries to assess different development models and efficiencies under varying policy and market conditions.
  (3) Impact of International Policies: Studying the effects of global policy changes on the promotion and acceptance of electric vehicles.
3. Application of the Framework Across Industries and Transport Modes:
  (1) Cross-Industry Applications: Applying the decision framework to the environmental assessment and market analysis of other modes of transport, such as aviation and railways.
  (2) Sustainable Transportation Systems: Supporting the design and optimization of sustainable transportation systems, evaluating the environmental and social impacts of different transportation strategies in urban planning.
  (3) Technology Transfer and Innovation: Investigating how the methodologies and tools from the electric vehicle evaluation framework can be transferred to other industries that require complex multi-attribute decision support, such as energy, manufacturing, and services.

These future research directions aim to advance the frontier of the Mo-RVIKOR method and its applications, providing broader and deeper decision support for the global electric vehicle industry and other fields.

## Supporting information

S1 Data (XLSX)
